# Transcriptome analysis of sugar beet in response to the pathogenic oomycete *Aphanomyces cochlioides*

**DOI:** 10.1186/s12870-024-05910-y

**Published:** 2024-12-18

**Authors:** Valentina Rossi, Louise Holmquist, Erik Alexandersson, Laura Grenville-Briggs

**Affiliations:** 1 Department of Plant Protection Biology, Swedish University of Agricultural Sciences, P.O. Box 190, Lomma, SE-234 22 Sweden; 2 DLF Beet Seed, Säbyholmsvägen 24, Landskrona, SE-261 91 Sweden; 3 Present Address: Nordic Beet Research, Borgeby Slottsväg 11, Bjärred, SE-237 91 Sweden; 4 Department of Plant Breeding, Swedish University of Agricultural Sciences, P.O. Box 190, Lomma, SE-234 22 Sweden

**Keywords:** *Beta vulgaris*, Aphanomyces root rot, Damping-off, Transcriptomics, Molecular plant-microbe interactions, Disease resistance

## Abstract

**Background:**

Aphanomyces root rot is one of the most severe diseases in sugar beet (*Beta vulgaris* L.), resulting in drastic losses in sugar yield and plant degeneration. The causal agent is the soil-borne pathogen *Aphanomyces cochlioides*, a phytopathogenic oomycete able to infect sugar beet roots from the seedling stage until harvest. Reliable control measures and fully resistant varieties to prevent the disease on mature roots are currently not available. Furthermore, the quantitative nature of the resistance mechanisms to the root rot disease remain unclear. With the aim to identify key genes involved in plant defense responses against the root rot, we performed a transcriptome analysis of sugar beet interactions with *A. cochlioides*. The transcriptome responses of two partially resistant and two susceptible sugar beet breeding lines, inoculated with three *A. cochlioides* isolates with different geographical origins have been investigated in this study.

**Results:**

The results showed that the transcriptional responses to *A. cochlioides* infection were mainly genotype-dependent. Comparisons of transcriptome profiles of partially resistant and susceptible breeding lines revealed the presence of differentially expressed genes that play a key role in defense mechanisms during the initial stages of infection. Gene Ontology (GO) categories associated with hydrogen peroxide (H_2_O_2_) metabolism, detoxification and cell wall organization were significantly enriched in the differentially expressed gene set from the two partially resistant lines, while photosynthesis-related GO terms were significantly enriched in the two susceptible lines. Unique and overlapping GO categories were over-represented in specific genotype-isolate-time point interactions, indicating that different genotypes respond with common defense strategies as well as specialized responses to different isolates and time points. Transcription factors belonging to the WRKY and ERF families were up-regulated in all genotypes. Furthermore, increased expression of genes encoding for disease resistant proteins have been identified in the two partially resistant genotypes.

**Conclusions:**

This research offers new insights into the transcriptomic events that regulate the sugar beet defense responses to *A. cochlioides* infection. The findings of this study emphasize the importance of genotype-specific interactions in response to different *A. cochlioides* isolates. Moreover, the results showed the up-regulation of genes that may play important roles in the defense responses to *A. cochlioides* which can be used to improve future breeding and to assist in the development of resistant cultivars.

**Supplementary Information:**

The online version contains supplementary material available at 10.1186/s12870-024-05910-y.

## Background

Sugar beet (*Beta vulgaris* ssp. *Vulgaris* L.) is an economically important crop that is cultivated for its high sucrose root content in temperate climates [1]. It provides nearly 30% of the annual sugar production worldwide. Moreover, during the sugar extraction process several by-products such as pulp and molasses are produced and used for animal feed and bioethanol production [2]. The development of varieties with high sugar yield is the main goal in sugar beet breeding programs. However, a multitude of secondary traits such as germination potential, bolting resistance and pest and disease resistances are required to fulfil the maximum yield [3]. High production standards are particularly threatened by pathogens which are responsible for yield losses and sugar reduction. One of the most problematic diseases in sugar beet is caused by the soil-borne pathogen *Aphanomyces cochlioides*. *A. cochlioides* is part of the Class oomycetes, fungal-like microorganisms able to infect a vast range of aquatic and terrestrial hosts and belongs to the Saprolegniales lineage [4, 5]. The life cycle of *Aphanomyces* spp consists of a sexual and an asexual stage. Thick-wall oospores are produced during the sexual phase, and they can persist in the soil up to ten years, representing a resilient source of infection [6]. In the asexual stage, vegetative hyphae differentiate into sporangia where biflagellate motile zoospores are produced. Zoospores are then released in the soil and swim chemiotactically towards the root surface, where they germinate, penetrate and lastly colonize the host tissue [7]. *A. cochlioides* is ubiquitous and it is specialized to infect members of the Amaranthaceae family including spinach (*Spinacia oleracea* L.) and cockscomb (*Celosia argentea* L.) [8] but it appears to be one of the main yield limiting factors in all sugar beet growing regions of the United States of America, Canada, Chile, Europe and Japan [9]. In sugar beet, the pathogen is the causal agent of an acute disease known as damping-off and a chronic root rot, which occur at the seedling stage and on mature roots, respectively [10]. The infection is initiated under warm and wet soil conditions and can lead to a loss of up to 100% in heavily infested fields when environmental factors are optimal for disease development. Although most losses would occur 1–3 weeks after emergence [1], the use of chemical fungicides on pelleted seeds is able to enhance protection towards seedling damping-off [11]. On the other hand, the application of chemicals is inadequate to control the chronic root rot, for which effective control measures are not available. Due to the persistence of the oospores in the soil, crop rotation cannot prevent disease outbreaks and other cultural practices are often insufficient in ensuring an adequate economic yield [12]. The introduction of resistance into sugar beet cultivars might therefore be the ultimate strategy to manage the chronic root rot. However, knowledge about the genetic background of the resistance to *A. cochlioides* is limited. Previous studies have demonstrated that the resistance to Aphanomyces root rot is transmitted in a dominant manner [13] and a genomic region responsible for disease resistance has been identified on chromosome III in the Japanese breeding line “NK-310 mm-O” [11]. Nonetheless, the additive effect of other smaller quantitative trait loci (QTLs) cannot be excluded and other aspects such as the position and the product of major genes involved in the defense responses are still unexplored. Plant responses to pathogens are complex. Therefore, a better understanding of the molecular mechanisms triggered during host-pathogen interaction is important for the implementation of control strategies. Transcriptome profiling using next generation sequencing technologies is a powerful tool to investigate and quantify changes in transcription levels in response to different conditions [14]. By comparing transcriptome changes, we can identify host-related genes and pathways that are involved in the defense mechanisms to pathogens. A recent transcriptome study on sugar beet has led to the identification of three genes encoding for major latex proteins (MLPs) with elevated transcription levels in partially resistant genotypes after infection with the soil-borne basidiomycete *Rhizoctonia solani* [15]. Here we used transcriptome analysis to study sugar beet responses upon infection with *A. cochlioides*. We generated a large set of transcript data by performing RNA-sequencing (RNA-seq) with the aim to identify differentially expressed genes in two partially resistant and two susceptible sugar beet breeding lines and to elucidate the molecular mechanisms that operate in response to the pathogen. Three different *A. cochlioides* isolates from different geographic origins (i.e., Sweden, USA and Japan) were used in this study and the specific interactions between the different sugar beet genotypes with the different *A. cochlioides* isolates were analyzed. The differentially expressed genes (DEGs) analysis primarily aimed to discover and characterize genes with a key role in defense responses for the future selection of candidate genes that can be used as target genes in genome editing and in marker assisted selection to support sugar beet breeding.

## Methods

### Plant material

Two partially resistant (G06, line nº 12052942 and G12, line nº 20014000) and two susceptible (G01, line nº 05012001 and G17, line nº 18012527) sugar beet breeding lines were used in this study. Seeds were provided by DLF Beet Seed, Landskrona, Sweden. The disease resistance indexes of genotypes G12 and G17, were published in a previous study [16]. In addition, the resistance indexes of genotypes G01 and G06 are shown here.

### *A. cochlioides* strains

Three *A. cochlioides* field isolates were used: Ac_Arhill2012, collected in Sweden (Tågarp, Skåne), Ac_01.7.6 collected in USA (Marshall, Minnesota) and Ac_Hokkaido01 collected in Japan (Hokkaido). The three isolates were single-spore isolated and cultivated by transferring a single zoospore on Corn Meal Agar (CMA) (Sigma-Aldrich, Saint Louis, MO, U.S.) media containing 0.005% chloramphenicol, to obtain single strains. *A. cochlioides* cultures were incubated at 21 °C in the dark and sub-cultured every 14 days in fresh CMA media. These isolates are known to be virulent and have been used in sugar beet breeding programs for many years to screen breeding material in the greenhouse. The identity of the three *A. cochlioides* isolates was confirmed in a previous study and all the isolates were proven to be virulent to sugar beet and to induce similar responses in susceptible and partially resistant lines [16].

### Production of *A. cochlioides* zoospores

CMA media with 14-day-old *A. cochlioides* cultures were cut into pieces and incubated in flasks containing 1,5 L of a sterile peptone solution (3 g/liter, pH = 7) at room temperature in the dark, to promote mycelial growth. After 7 days the peptone solution was removed, and the agar pieces and the mycelium produced were incubated in 3 L of 2 mM NaCl ddH2O to induce sporulation. The solution was aerated overnight by pumping air through sterile glass pipettes connected to an aquarium pump. The agar pieces and the mycelia were drained and discarded while the zoospores released into the solution were counted in a hemocytometer.

### Plants growth and inoculation for disease resistance index

To assess the relationship between the genotypes and the different *A. cochlioides* isolates, plants were grown and inoculated in a climate chamber as described in Rossi et al. [16]. In brief, 12-week-old plants were inoculated by watering the soil with 150 ml of zoospores solution (5–7 × 10^4^ zoospores/ml), obtained from the three different isolates, separately. Plants were kept under controlled conditions (16 h light and 8 h dark, 22 °C day/night, 95% RH, 400 ppm CO2) and scored 20 days after the inoculation using a scoring scale of 5 classes: 1 = dead plants, 3 = severe infection, with fully necrotic hypocotyl, 5 = medium infection, only 50% of the hypocotyl is necrotic, 7 = mild infection, only the root tip is necrotic, 9 = no infection. For each genotype, 15 plants inoculated with each *A. cochlioides* isolate and 15 plants inoculated with water were collected from one independent experiment. A two-way ANOVA test with interactions, followed by a Tukey HSD (Honestly Significant Difference), test was conducted with the R-package tidyverse in R (version 4.0.5) to assess statistical significance between genotype- *A. cochlioides* isolate interactions.

### Plants growth and inoculation with *A. cochlioides* for RNA sequencing

**Fig. 1:**
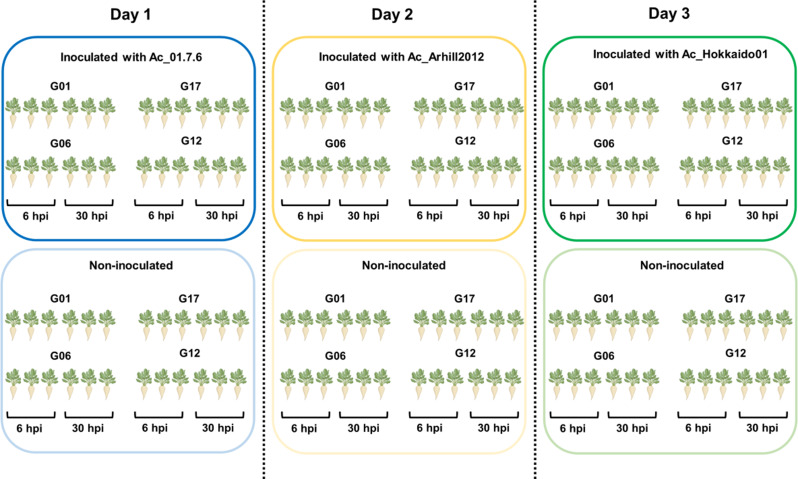
RNA-seq experimental set-up. 6-week-old sugar beet plants belonging to 4 different genotypes (G01, G17, G06 and G12) were inoculated with *A. cochlioides* zoospores once, in three different days, each day using a different *A. cochlioides* isolate (Ac_01.7.6, from USA, Ac_Arhill2012, from Sweden and Ac_Hokkiado01, from Japan). Roots were collected at 6 hpi and 30 hpi. Control plants, inoculated with 2 mM NaCl water, were collected for each *A. cochlioides* isolate, at the same time point (6 hpi and 30 hpi). Three biological replicates consisting of a single plant root/replicate were collected for both inoculated and non-inoculated plants for a total of 144 samples

To minimize any possible contamination, seeds were surface disinfected by submersion in deionized water for 30 min followed by submersion for 5 min in a 56 °C water bath, briefly submerged in cold water and dried at room temperature for one day. Seeds were then germinated in steam-sterilized sowing soil in pots (ø =12 cm) and placed in a greenhouse. One week after emergence, seedlings were transplanted to new pots, containing 4 seedlings each, and grown in the greenhouse for 3 weeks. Plants were then transferred to a climate chamber and grown under controlled conditions (16 h light and 8 h dark, 22 °C day/night, 95% RH, 400 ppm CO2) for two weeks. For inoculation, six-week-old plants were harvested, rinsed and placed on a rack obtained from the top of a 10 ml-tip box and mounted on the box filled with water for 48 h, to submerge the roots. In order to induce infection, the water in the box was then replaced with *A. cochlioides* zoospores solution (5–7 × 10^4^ zoospores/ml) and roots were incubated in the inoculum for 2 h. This time was shown to be sufficient for the pathogen to reach the plant, encyst and penetrate the root [16]. The inoculum was then removed and replaced with water, where roots were submerged until harvesting. Roots were collected at 6 and 30 h post inoculation (hpi), snap frozen in liquid N_2_ and stored at -80℃ until further use. Roots belonging to the four different genotypes submerged in 2 mM NaCl ddH_2_O instead of the zoospore solution were used as non-inoculated samples at each time point. Three biological replicates consisting of a single root from an individual plant each were collected for both treated and untreated plants. Inoculation of the plants was performed once, every fourth day, on three different days, each day using a different *A. cochlioides* isolate and non-inoculated samples were collected in each of the three inoculation experiments (Fig. 1).

However, not all non-inoculated samples were used in the RNA sequencing, therefore in the differential gene expression analysis, some inoculated samples were compared to untreated samples collected on a different day but at the exact same time of the light cycle. Table 1 shows the pairwise comparisons between the inoculated and non-inoculated samples that were included in the transcriptome analysis, for a total of 120 samples.

**Table 1:** List of inoculated and non-inoculated samples included in the transcriptome analysis. Table shows the genotype (G01, G17, G06 or G12), time point (6 hpi or 30 hpi) and *A.cochlioides* isolate (Ac_01.7.6, Ac_Arhill2012 or Ac_Hokkaido01) used. In the non-inoculated samples, the name of *A. cochlioides* isolate indicates whether the samples were collected during the inoculation with the isolate from USA (Ac_01.7.6), Sweden (Ac_Arhill2012) or Japan (Ac_Hokkaido01), followed by NI (Non-Inoculated). Three biological replicates per each sample were collected, producing a total of 120 samples

Inoculated			Non-inoculated	
Genotype	Time point	Isolate		Isolate
G01	6hpi	Ac_01.7.6	vs.	Ac_01.7.6 _NI
G01	6hpi	Ac_Arhill2012	vs.	Ac_Arhill2012 _NI
G01	6hpi	Ac_Hokkaido01	vs.	Ac_Hokkaido01 _NI
G01	30hpi	Ac_01.7.6	vs.	Ac_01.7.6 _NI
G01	30hpi	Ac_Arhill2012	vs.	Ac_Arhill2012 _NI
G01	30hpi	Ac_Hokkaido01	vs.	Ac_Hokkaido01 _NI
G17	6hpi	Ac_01.7.6	vs.	Ac_Arhill2012 _NI
G17	6hpi	Ac_Arhill2012	vs.	Ac_Arhill2012 _NI
G17	6hpi	Ac_Hokkaido01	vs.	Ac_Arhill2012 _NI
G17	30hpi	Ac_01.7.6	vs.	Ac_Arhill2012 _NI
G17	30hpi	Ac_Arhill2012	vs.	Ac_Arhill2012 _NI
G17	30hpi	Ac_Hokkaido01	vs.	Ac_Arhill2012 _NI
G06	6hpi	Ac_01.7.6	vs.	Ac_01.7.6 _NI
G06	6hpi	Ac_Arhill2012	vs.	Ac_Arhill2012 _NI
G06	6hpi	Ac_Hokkaido01	vs.	Ac_Hokkaido01 _NI
G06	30hpi	Ac_01.7.6	vs.	Ac_01.7.6 _NI
G06	30hpi	Ac_Arhill2012	vs.	Ac_Arhill2012 _NI
G06	30hpi	Ac_Hokkaido01	vs.	Ac_Hokkaido01 _NI
G12	6hpi	Ac_01.7.6	vs.	Ac_Arhill2012 _NI
G12	6hpi	Ac_Arhill2012	vs.	Ac_Arhill2012 _NI
G12	6hpi	Ac_Hokkaido01	vs.	Ac_Arhill2012 _NI
G12	30hpi	Ac_01.7.6	vs.	Ac_Arhill2012 _NI
G12	30hpi	Ac_Arhill2012	vs.	Ac_Arhill2012 _NI
G12	30hpi	Ac_Hokkaido01	vs.	Ac_Arhill2012 _NI

### RNA extraction, library preparation and sequencing

Roots were ground to a fine powder in frozen grinding jars in a TissueLyser II (QIAGEN) and total RNA was extracted from approximately 100 mg of ground tissue using RNAqueous™-4PCR Total RNA Isolation Kit (Invitrogen), according to the manufacturer’s instructions. RNA integrity was assessed by running aliquots of 2 µl in an RNA 6000 Nano LabChip (Agilent Technologies, CA, USA) in the 2100 Bioanalyzer (Agilent Technologies, CA, USA) and samples with a RNA integrity number (RIN) > 8 were used. cDNA libraries were constructed from mRNA selected through poly-A enrichment using a TruSeq^®^ Stranded mRNA Library Prep Kit (Illumina, CA, USA) and sequenced in the Illumina NovaSeq 6000 platform (SciLifeLab, Stockholm, Sweden). A quality control was performed on the generated paired-end mRNA reads with FastQC [17]. Contaminant adapters and low quality reads were removed using the wrapper script Trim Galore v.0.6.6 [18] and paired-end clean reads were aligned to the sugar beet reference genome EL10 [19] and to the *A. cochlioides* reference genome UMN_Aphcoc_1.0 [20] using STAR software v.2.7.0a [21] with default parameters and the mapped BAM files were used in featureCounts software v. 2.0.3 [22] to generate raw read counts.

### Differential gene expression analysis

Principal Component Analysis (PCA) was performed using the ggplot2 R package and read count normalization and differential gene expression analysis to compare inoculated and uninoculated samples at the same time point were conducted using the DESeq2 package [23]. In DESeq2, the p-values obtained by the Wald test are corrected for multiple testing using the Benjamini and Hochberg [24] method by default. Genes were considered to be significantly differentially expressed if their expression levels had a false discovery rate (FDR) value < 0.05 and a |log2 fold change| >1. Venn diagrams were created using Venny v2.1.0 [25] to assess overlapping DEGs between different experimental groups. Differential expression analysis was conducted in individual contrasts according to genotype, time point and *A. cochlioides* isolate with the respective non-inoculated sample, separately.

### Gene Ontology (GO) enrichment and Kyoto Encyclopedia of genes and genomes (KEGG) analysis

To obtain functional annotations, all sugar beet transcripts from the reference genome EL10 were blasted against *Arabidopsis thaliana* genome from the *A. thaliana* database TAIR10, released on 2019-07-11, using BLASTp. Gene Ontology (GO) enrichment and KEGG pathways analysis were performed using ShinyGO v0.77 [26] to identify the most significant enriched GO terms and pathways (FDR < 0.05, calculated based on nominal p-value from the hypergeometric test), using *A. thaliana* as reference species. Shared and unique GO biological processes were visualized in chord diagrams created using the R package circlize [27].

### Transcription factor (TF) enrichment analysis

To gain insight into the upstream regulators among the differentially expressed genes (DEGs) in different genotypes, a transcription factor (TF) analysis was performed using the *A. thaliana* TF list from Plant Transcription Factor Database v5.0 (PlantTFDB) (http://planttfdb.gao-lab.org/index.php?sp=Ath) [28]. Genes with a |log2 fold change| > 0 and FDR < 0.05 were used as input genes.

## Results

### Disease resistance indexes from the phenotypic evaluation

All three *A. cochlioides* isolates used in this study were virulent on sugar beet plants, and different genotypes showed consistent levels of resistance or susceptibility against the three strains. In a previous study, genotypes G12 and G17 were classified as partially resistant and susceptible, respectively (Table 2). As a complement to this data, the disease resistance indexes for genotypes G01 and G06 are also shown in Table 2. There was no significant difference in virulence between isolates within a single genotype.

**Table 2:** Disease resistance indexes. Average scores of different sugar beet genotypes (G01, G17, G06, G12) in response to different *A. cochlioides* isolates (Ac_USA01.7.6, Ac_Arhill2012, Ac_Hokkaido01,). 15 biological replicates for each genotype were collected to calculate the average scores

	G01	G17	G06	G12		
Ac_01.7.6		1.93 a	1.00 a	6.60 b	6.73 b	
Ac_Arhill2012		1.66 a	1.27 a	6.60 b	6.80 b	
Ac_Hokkaido01		1.93 a	1.40 a	6.47 b	6.27 b	

### Mapping of reads to the sugar beet reference genome

To study the influence of *A. cochlioides* infection on sugar beet gene expression, the host transcript data obtained by RNA-seq from uninfected and infected roots were compared. Transcriptome sequencing generated between 21 and 171 million reads per sample with a length of 151 bp, only one sample (G01_30hpi_Hokkaido_01, replicate 3) had a lower number of reads (14 million). After quality inspection and filtering to remove adapter sequences and low-quality reads, between 20 and 170 million high quality reads with a GC content of 42–45% were obtained. 84–91% of the reads mapped to the sugar beet reference genome EL10 [19] (Table 3) and mapping levels were similar between genotypes indicating that little genotype-bias was introduced using the EL10 reference genome for all genotypes. Less than 1% of the generated reads from infected samples mapped to the *A. cochlioides* reference genome UMN_Aphcoc_1.0 [20], indicating a limited number of pathogen transcripts in the samples, while 0% of the reads mapped to *A. cochlioides * from the non-inoculated samples, as expected.

**Table 3:** Transcriptome statistics of sugar beet reads. Total number of reads (million) and percentage of reads mapping to the sugar beet reference genome for all the samples at 6 and 30 h post inoculation (hpi), average of three biological replicates

	Sugar beet lines							
	G01		G17		G06		G12	
Time point (hpi)	6	30	6	30	6	30	6	30
Total number of reads (Million)	32.99	45.51	46.25	51.53	44.73	42.28	50.38	46.69
Mapping reads to sugar beet reference genome EL10 (%)	85.4	87.5	88.1	85.2	86.8	86.2	87.4	87.8

### Principal component analysis and sample clustering

**Fig. 2:**
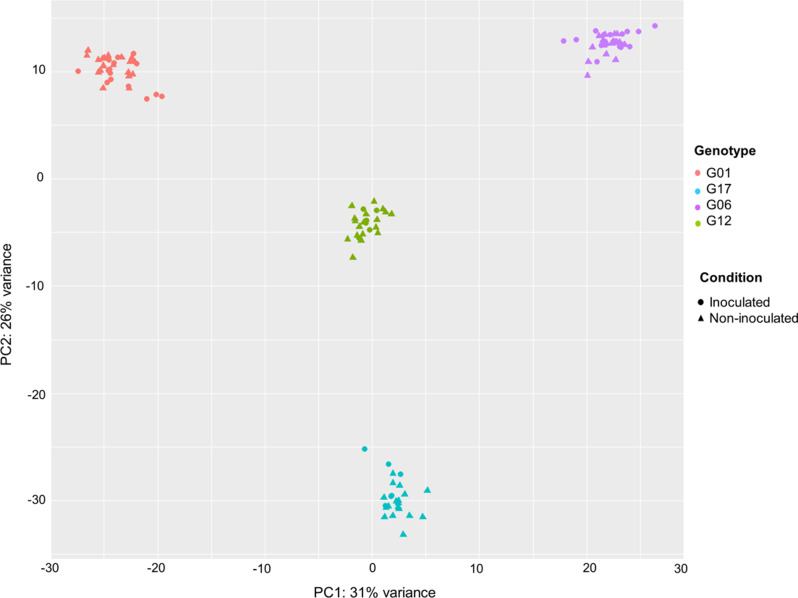
Principal Component Analysis (PCA) plot showing samples inoculated with three different *A. cochlioides* isolates (Ac_01.7.6, Ac_Arhill2012, Ac_Hokkaido01), at 6 hpi and 30 hpi, and non-inoculated samples, that cluster together according to the genotypes to which they belong

Before performing differential expression (DE) analysis, a Principal Component Analysis (PCA) was performed to explore variation between samples. After performing PCA, one sample (G01_6hpi_Ac_Hokkaido01_NC, replicate 3), was concluded to be an outlier and it was therefore removed prior to DE analysis. Overall, samples clustered together based on their genotype (Fig. 2), while other variables (i.e. inoculation, time point, *A. cochlioides* isolate) represent a more minor source of variation in the dataset. 3D PCA plots were realized to observe variation between samples used for individual pairwise comparisons (Additional files 1–12). Hierarchical clustering of transformed data was visualized as a heatmap at different timepoints (Additional files 13 and 14).

### Differentially expressed genes in sugar beet during infection

In the DE analysis, a total of 4262 differentially expressed genes (DEGs) were identified. The number of DEGs responding to the treatment in the susceptible line G01 was 1987, where 1149 transcripts were up-regulated and 838 were down-regulated. In the susceptible line G17 the total number of DEGs was 2013, of which 1011 were up-regulated and 1002 were down-regulated. In total, 547 transcripts were differentially expressed in the partially resistant line G06 compared to the control, of which 293 were up-regulated while 254 displayed a down-regulation. In the partially resistant genotype G12 a total of 2292 DEGs were observed. Of these, 999 were up-regulated and 1293 were down-regulated. The number of DEGs that were up- and down-regulated in different genotypes in response to different *A. cochlioides* isolates at different time point is summarized in Table 4.

**Table 4:** Number of up- and down-regulated genes in different genotypes in response to different *Aphanomyces cochlioides* isolates at 6 hpi and 30 hpi

Genotype	Isolate	Time point			
		6 hpi		30 hpi	
		Up-regulated	Down-regulated	Up-regulated	Down-regulated
	Ac_01.7.6	678	421	126	82
G01	Ac_Arhill2012	36	9	73	98
	Ac_Hokkaido01	197	158	280	199
	Ac_01.7.6	167	185	291	428
G17	Ac_Arhill2012	10	1	369	133
	Ac_Hokkaido01	223	245	444	444
	Ac_01.7.6	16	12	178	56
G06	Ac_Arhill2012	39	30	10	72
	Ac_Hokkaido01	30	68	36	32
	Ac_01.7.6	114	112	363	653
G12	Ac_Arhill2012	117	111	131	299
	Ac_Hokkaido01	132	156	671	757

**Fig. 3:**
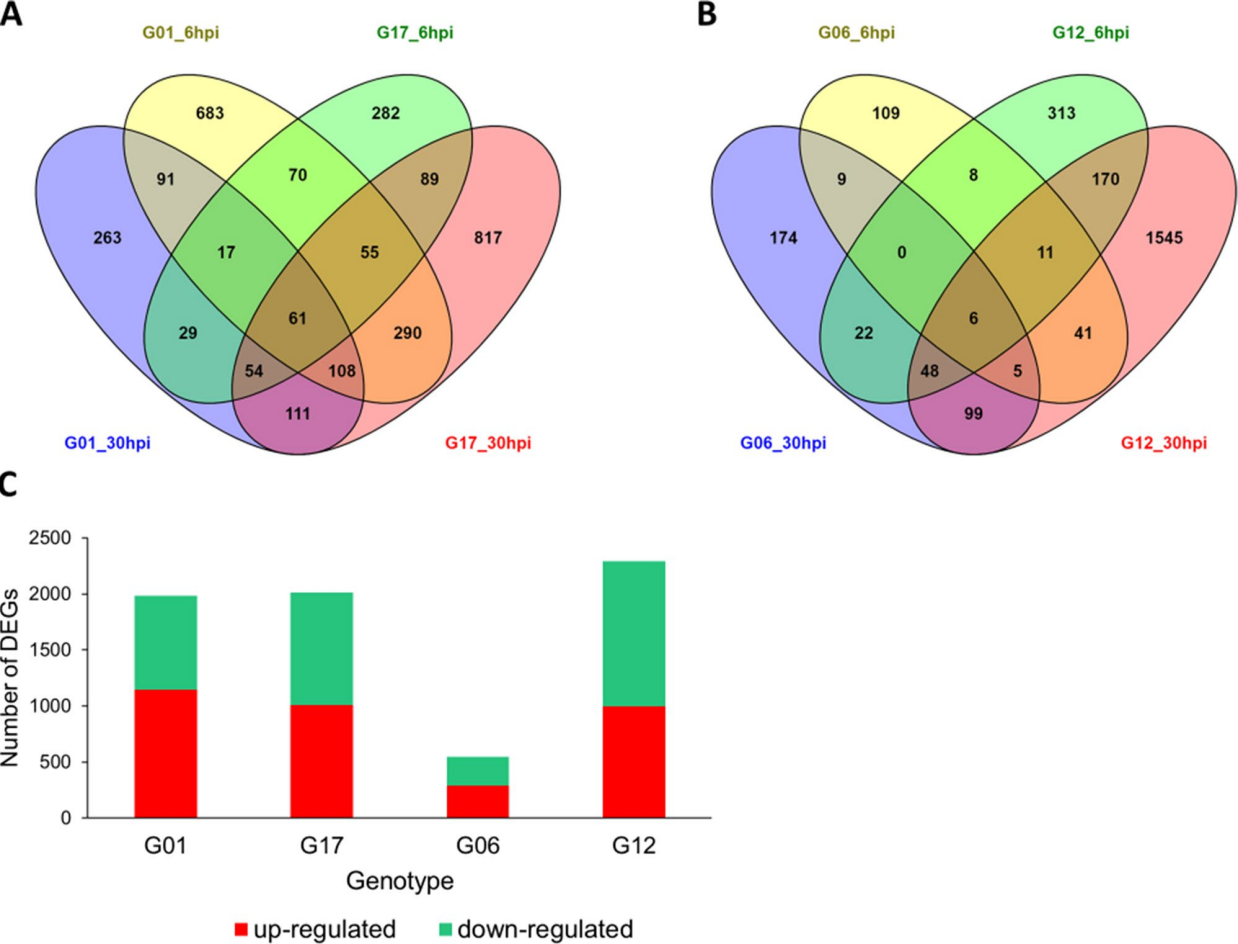
Number of differentially expressed genes (DEGs) in sugar beet lines in response to three different *Aphanomyces cochlioides* isolates. Venn diagram showing the total number of DEGs uniquely expressed or shared between samples in **(A)** the two susceptible genotypes G01 and G17 and **(B)** the two partially resistant genotypes G06 and G12, at 6 and 30 h after inoculation with three different *A. cochlioides* isolates. **(C)** Bar chart showing the total number of up- and down-regulated DEGs in all genotypes

A total of 111 DEGs were shared between all the genotypes tested, including chalcone synthases (CHS), auxin-binding proteins, glutathione S-transferases (GSTs) and germin-like proteins (GLP). A total of 1242 transcripts were differentially expressed uniquely in the two partially resistant genotypes and 1702 DEGs were exclusively represented in the susceptible genotypes. The number of DEGs in susceptible and partially resistant genotypes that were unique or that overlapped between the two time points is shown in Fig. 3. Changes in transcript level increased over time, with a higher number of DEGs at 30 hpi compared to the earliest time point in all sugar beet lines tested, except for the susceptible genotypes G01 in response to Ac_01.7.6 and G06 in response to Ac_Hokkaido01 that showed a higher number of DEGs at 6 hpi.

### Principal component analysis and differentially expressed genes in non-inoculated sugar beet genotypes

Since genotype appeared to be the major source of variation between samples, a principal component analysis was also performed using the non-inoculated samples datasets, to explore the variation between different genotypes, prior to the infection. Samples clustered together according to the genotypes to which they belong (Additional file 15). A total of 3484 genes were differentially expressed in the partially resistant genotype G06 compared to the susceptible genotype G01. In particular, 1829 genes were up-regulated, while 1655 were down-regulated. The number of DEGs in genotype G06 compared to the susceptible genotype G17 was 3187, of which 1815 were up-regulated and 1372 were down-regulated. A total of 2689 DEGs were identified in the partially resistant genotype G12 compared to genotype G01, with 1500 genes being up-regulated and 1189 genes being down-regulated. The total number of DEGs in genotype G12 compared to genotype G17 was 2531, where 1505 were up-regulated and 1026 were down-regulated.

### Enriched GO biological processes and KEGG pathways in different sugar beet genotypes in response to *A. cochlioides* infection

**Fig. 4:**
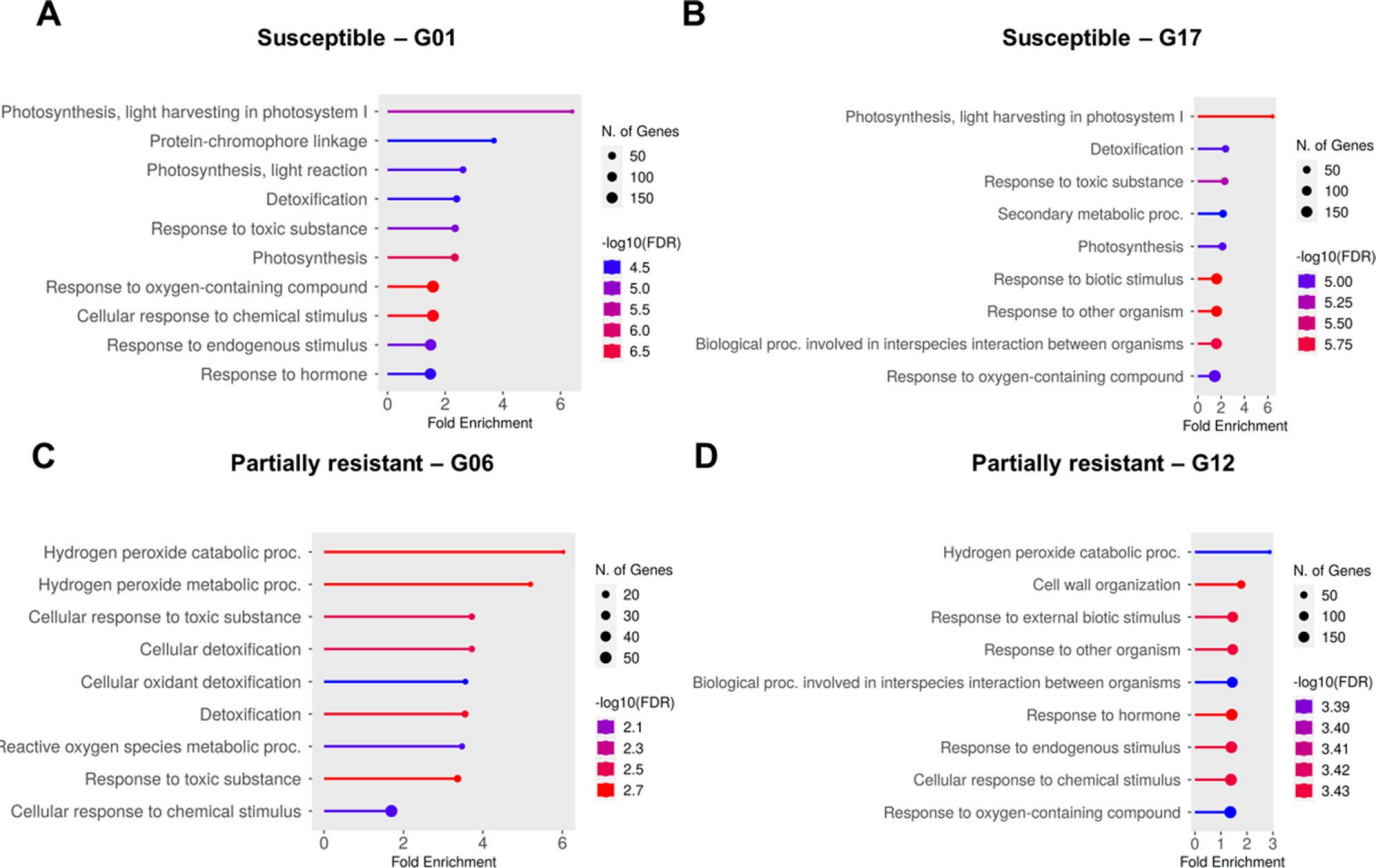
Biological processes of the most significantly enriched (FDR < 0.05) GO terms from all the up- and down-regulated genes in susceptible and partially resistant sugar beet lines. Charts showing the ten most significant GO biological processes in the sugar beet susceptible lines **(A)** G01 and **(B)** G17 and in the partially resistant lines **(C)** G06 and **(D)** G12

To better understand the biological processes contributing to sugar beet resistance and susceptibility against *A. cochlioides*, a Gene Ontology (GO) term enrichment analysis was performed on up- and down-regulated genes in all sugar beet lines evaluated in response to the three different *A. cochlioides* isolates. The biological processes of the ten most enriched GO terms from DEGs for each genotype are illustrated in Fig. 4.

The GO terms carbon fixation, flavonoid metabolism, response to toxic substance/detoxification and photosynthesis had a fold enrichment > 2 in genotype G01 at 6 hpi. Photosynthetic processes, especially involved in the light reaction were the most represented biological processes at 30 hpi.

Hydrogen peroxide (H_2_O_2_) metabolism, detoxification/response to toxic substance processes and secondary metabolites were the most enriched biological processes in G17 at 6 hpi, while photosynthesis, response to toxic substance, response to fungus and interspecies interaction were the most enriched at 30 hpi.

Overall, GO terms for biological processes involved in the photosynthetic activity and detoxification were significantly enriched in the two susceptible sugar beet lines G01 and G17 (Figs. 3B and 4A). GO enrichment analysis on the set of DEGs that were uniquely expressed in the two susceptible genotypes showed a predominance of gene changes in the photosynthesis process (“Photosynthesis, light reaction”, “Photosynthesis”), in response to decreased oxygen levels and in the glucose and carbohydrate metabolic processes.

In the partially resistant line G06, the GO terms reactive oxygen species (ROS) metabolic processes, particularly H_2_O_2_ metabolism and detoxification processes were found to be significantly enriched, with a fold enrichment > 2 (Fig. 4C). These GO biological processes were mostly represented at 30 hpi.

Overall, in the partially resistant line G12, diverse enriched GO biological processes were found, including cell wall organization, responses to other organism and biotic stimulus, interspecies interaction between organisms and response to hormone, all with a fold enrichment < 2, except for “H_2_O_2_ catabolic process” that showed a fold enrichment of 2.9 (Fig. 4D). GO biological processes involved in the ROS and H_2_O_2_ metabolism, detoxification and in the microtubule-based movement showed a fold enrichment > 2 at 6 hpi, while “Response to chitin” and “cell wall organization” processes showed a fold enrichment > 2 at 30 hpi.

GO biological processes in common between the two partially resistant lines were “H_2_O_2_ catabolic process” and “Cellular response to chemical stimulus”.

**Fig. 5:**
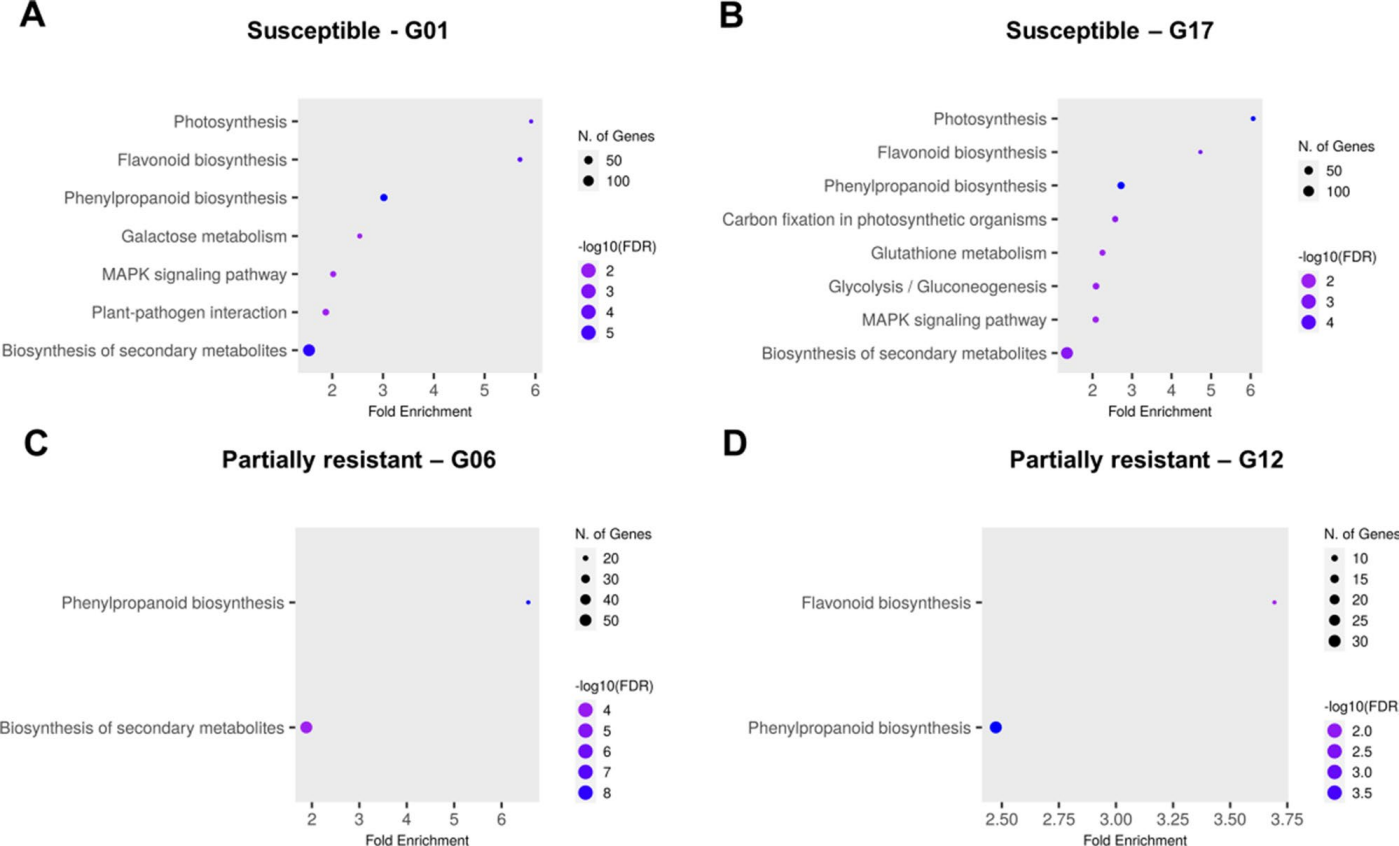
KEGG enriched pathways from differentially expressed genes from **(A)** the susceptible genotype G01 dataset; **(B)** the susceptible genotype G17 dataset; **(C)** the partially resistant genotype G06 dataset and **(D)** the partially resistant genotype G12 dataset

Common enriched pathways in the two susceptible genotypes were photosynthesis, flavonoid and phenylpropanoid biosynthesis, MAPK signaling pathway and biosynthesis of secondary metabolites. KEGG enriched pathways in the partially resistant genotypes included flavonoid, phenylpropanoid and secondary metabolite biosynthesis (Fig. 5).

### Enriched GO biological processes in different sugar beet genotypes in response to different *A. cochlioides* isolates and time points

GO enrichment analysis of up- and down-regulated genes showed biological processes uniquely represented in specific genotype-isolate-time point interactions as well as common biological processes shared among different genotypes in response to different isolates and time points. Shared responses were mainly over-represented between genotypes G01 and G06 in response to Ac_Arhill2012 and Ac_Hokkaido01 at 6 hpi and were related to photosynthesis-related processes. In addition, genotypes G17 and G12 shared several GO biological processes related to detoxification and toxin catabolic processes in response to Ac_Arhill2012 and Ac_Hokkaido01 at different time points.

### Response to Ac_01.7.6

**Fig. 6:**
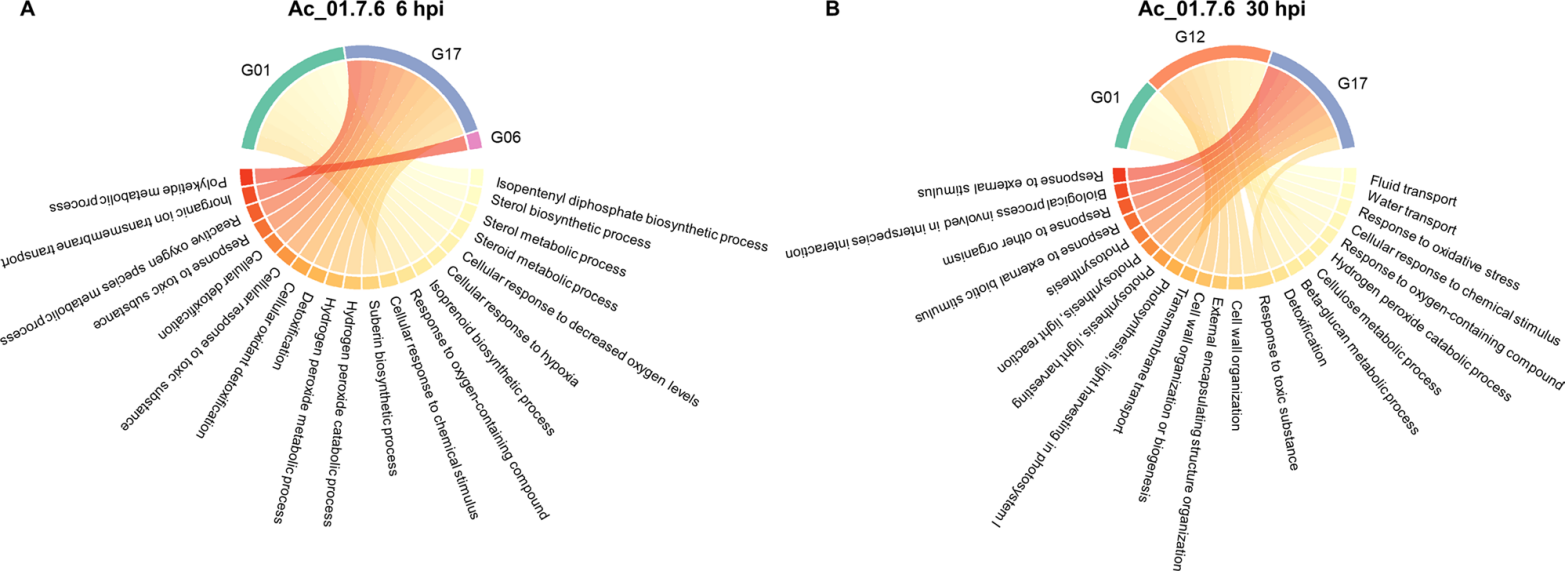
Top 10 Gene Ontology (GO) biological processes in DEGs of susceptible (G01 and G17) and partially resistant (G06, G12) sugar beet lines is response to the *A. cochlioides* isolate Ac_01.7.6 at **(A)** 6 hpi and **(B)** 30 hpi

Unique responses were observed in different genotypes in relation to Ac_01.7.6 at both time points (Fig. 6). At 6 hpi, genotype G01 had GO terms related to sterol metabolism, isoprenoid biosynthesis, and responses to oxygen and chemical stimuli. Genotype G17 showed GO terms related to processes like detoxification, H_2_O_2_ metabolism, and response to toxic substances. Genotype G06 had a single unique process, related to polyketide metabolism. No enriched GO terms were found in genotype G12 at this time point. At 30 hpi, enriched GO terms in genotype G01 were related to transport processes, oxidative stress response, and responses to chemicals and oxygen-containing compounds. In genotype G17 primarily enriched GO terms were photosynthesis-related processes, responses to biotic stimuli, and interspecies interactions, while in G12 enriched GO processes were H_2_O_2_ catabolism, cell wall organization, cellulose and beta-glucan metabolism, and detoxification. No enrichment was found in genotype G06 at 30 hpi from the total DEGs. However, GO enrichment analysis on up-regulated genes showed an enrichment in proline metabolic process, H_2_O_2_ and polysaccharide catabolism, reactive oxygen species metabolism and response to toxic substance.

### Response to Ac_Arhill2012

**Fig. 7:**
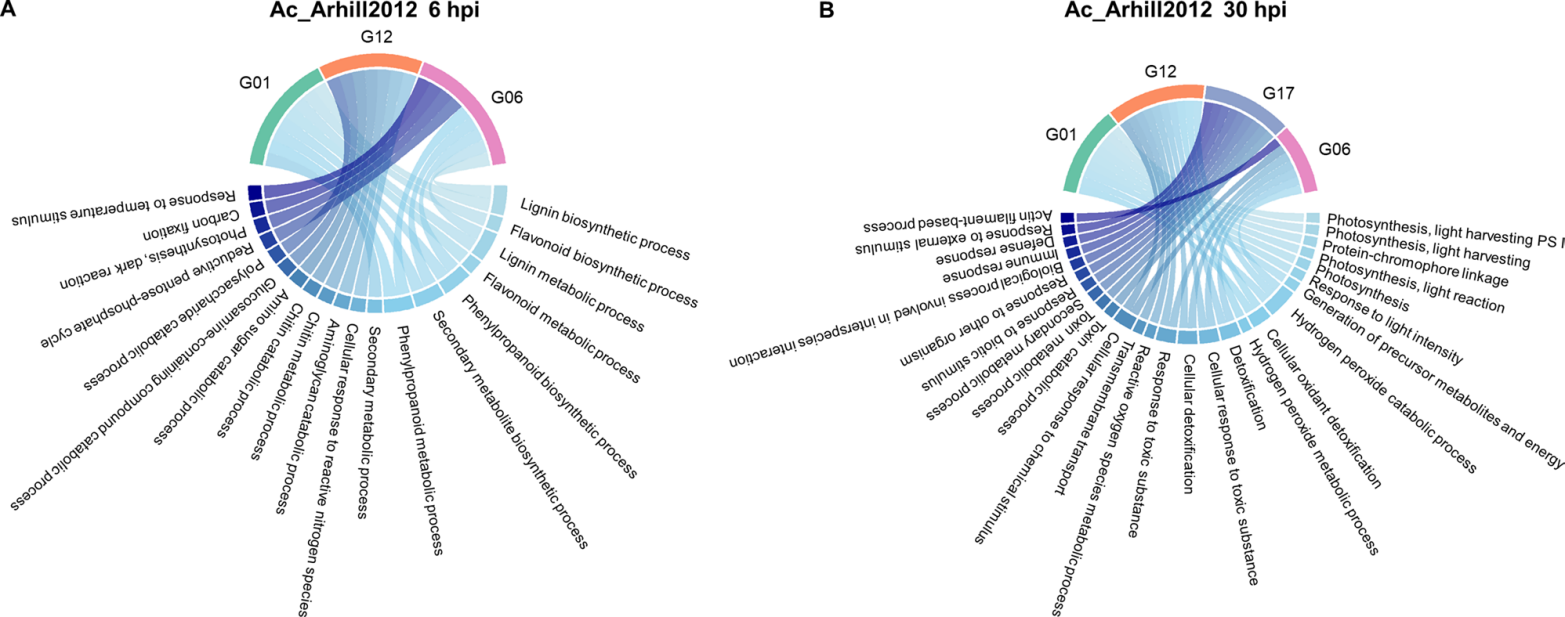
Top 10 Gene Ontology (GO) biological processes in DEGs of susceptible (G01 and G17) and partially resistant (G06, G12) sugar beet lines is response to the *A. cochlioides* isolate Ac_Arhill2012 at **(A)** 6 hpi and **(B)** 30 hpi

GO enrichment analysis showed unique and common GO terms in different genotypes. At 6 hpi, enriched GO biological processes in G01 were related to lignin and flavonoid biosynthesis and other secondary metabolites. Enriched GO biological processes in G12 were catabolism of chitin, aminoglycans, and polysaccharides, and there were no common processes with other genotypes. Enriched GO biological processes in genotype G06 were related to the photosynthesis and carbon fixation processes. This genotype shared several processes related to lignin, phenylpropanoid biosynthesis and secondary metabolites with G01. No enriched GO terms were found in genotype G17 at 6 hpi (Fig. 7A). At 30 hpi, enriched GO biological processes in G01 were related to photosynthesis and light harvesting. Unique enriched GO in G12 were related to detoxification processes and responses to toxic substances. Common processes in H_2_O_2_ catabolism and cellular oxidant detoxification were shared with G12 and G06. In G17, enriched biological processes were related to immune and defense responses and responses to biotic stimulus and to other organism, as well as secondary metabolic processes, with no common processes with the other genotypes. Enriched GO biological processes in G06 were involved in cellular detoxification, actin filament-based process, cellular response to toxic substance, response to toxic substance and cellular response to chemical stimulus (Fig. 7B).

### Response to Ac_Hokkaido01

**Fig. 8:**
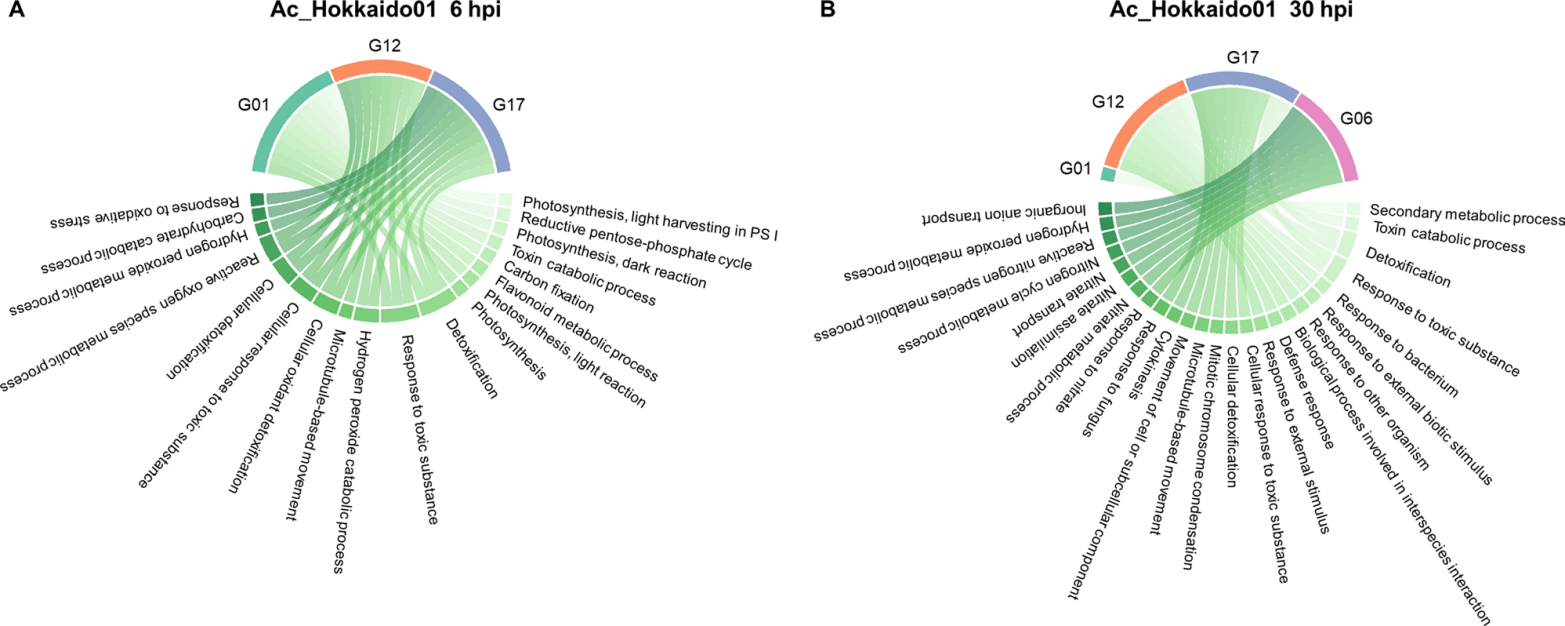
Top 10 Gene Ontology (GO) biological processes in DEGs of susceptible (G01 and G17) and partially resistant (G06, G12) sugar beet lines is response to the *A. cochlioides* isolate Ac_Hokkaido01 at **(A)** 6 hpi and (B) 30 hpi

Unique responses were also observed in response to Ac_Hokkaido01. However, genotypes G12 and G17 showed commonly represented GO biological processes at both time points (Fig. 8). At 6 hpi, enriched GO process in G01 were unique and associated with many photosynthesis-related processes and flavonoid metabolism. Genotypes G12 and G17 shared many processes related to oxidative stress response, detoxification, and H_2_O_2_ metabolism. Genotype G17 had some unique processes related to carbohydrate metabolism and response to oxidative stress. No enriched GO terms were found in genotype G06 at 6 hpi from total DEGs. However, enriched GO terms from the up-regulated genes were related to toxin catabolic and metabolic processes, glutathione metabolic processes, response to wounding and secondary metabolic processes. At 30 hpi, genotype G01 had a unique association with secondary metabolic processes. Genotype G12 was characterized by various responses to biotic stimuli and defense mechanisms, which were not present in the other genotypes. Genotype G17 had unique processes related to cell movement, cytokinesis, and responses to fungi. Genotype G12 and G17 shared some enriched GO biological processes such as detoxification, response to toxic substance, cellular response to toxic substance, and cellular detoxification. Genotype G06 was uniquely associated with nitrate metabolism and nitrogen cycle processes.

### Transcription factor analysis

**Fig. 9:**
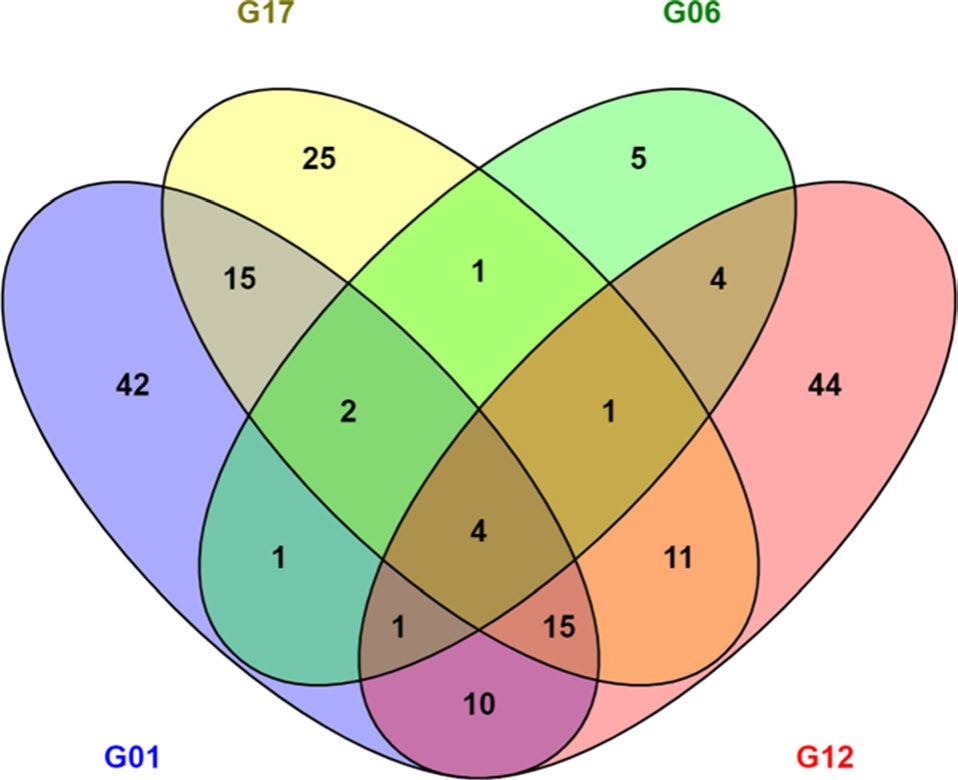
Number of up- and down-regulated transcription factors (TFs) in the four sugar beet genotypes analyzed

A total of 181 transcription factors (TFs) were identified among the significantly up- and down-regulated genes, 90 and 74 of which were differentially expressed in the susceptible genotypes G01 and G17, respectively, while 19 and 90 were detected in the DEGs of the two partially resistant lines G06 and G12, respectively (Fig. 9; Table 5).

**Table 5:** Number of up- and down-regulated transcription factors (TFs) at 6 hpi and 30 hpi in different genotypes

Genotype	Number of up-regulated TFs		Number of down-regulated TFs	
	6 hpi	30 hpi	6 hpi	30 hpi
G01	37	20	32	12
G17	8	39	12	25
G06	4	3	5	7
G12	10	47	9	30

Several TFs known to be involved in resistance to pathogens were up-regulated at either or both time points. They belong to the ERF (ERF1A, ERF8, ERF095, ERF109, RAP2-3), WRKY (WRKY40, WRKY70) and HB (OCP3) families (Table 6).

**Table 6:** Transcription factors (TFs) up-regulated in response to *A. cochlioides* in different genotypes

Transcript ID	Log_2_ fold change	Genotype	Time point	Isolate	Name	Description
EL10Ac6g15753	5.09 2.04	G01 G12	6 hpi 30 hpi	Ac_01.7.6 Ac_Hokkaido01	ERF109	Defense response to fungus
EL10Ac7g17111	1.97 2.19 2.78	G01 G12 G17	6 hpi 30 hpi 30 hpi	Ac_01.7.6 Ac_01.7.6 Ac_01.7.6	WRKY70	Defense response to bacterium, fungus and oomycetes
EL10Ac7g17571 EL10Ac3g06658	1.30 1.00	G01 G06	30 hpi 6 hpi	Ac_01.7.6 Ac_Arhill2012	RAP2-3 RAP2-3	Binds to the GCC-box pathogenesis-related promoter element, response to other organism
EL10Ac5g12314	1.15 1.75 1.38	G01 G12 G17	30 hpi 30 hpi 30 hpi	Ac_Hokkaido01 Ac_01.7.6 Ac_01.7.6	ERF1A	Defense response, response to nematode
EL10Ac9g22209	1.01 1.91 1.18	G01 G12 G17	30 hpi 30 hpi 30 hpi	Ac_Hokkaido01 Ac_01.7.6 Ac_Arhill2012	WRKY40	Regulation of defense response to bacterium and fungus
EL10Ac4g08236	0.71 1.05 1.29 1.55	G12 G12 G17 G17	6 hpi 30 hpi 6 hpi 30 hpi	Ac_Hokkaido01 Ac_Hokkaido01 Ac_Arhill2012 Ac_Hokkaido01	ERF8	Binds to the GCC-box pathogenesis-related promoter element
EL10Ac3g05025	1.06	G17	30 hpi	Ac_01.7.6	ERF095	Binds to the GCC-box pathogenesis-related promoter element
EL10Ac2g02609	0.75	G12	30 hpi	Ac_01.7.6	OCP3	Induced systemic resistance, regulation of defense response by callose deposition

### Up-regulated genes in the partially resistant sugar beet genotypes

Since we aimed to identify candidate genes playing a role in the defense mechanisms to *A. cochlioides*, genes that were uniquely up-regulated in the partially resistant genotypes were examined. Among the 18 up-regulated genes shared between genotypes G06 and G12, the transcript EL10Ac7g18219 annotated in SWISS-PROT as “Probable disease resistance protein At1g15890” was up-regulated at 30 hpi in both genotypes, with a log2 fold change of 2.75 in G12 in response to Ac_Hokkaido01 and 1.62 in G06 in response to Ac_01.7.6 and the transcript EL10Ac5g12824, annotated in SWISS-PROT as “Mitogen-activated protein kinase 4” and expressed in the plant-pathogen interaction pathway, was up-regulated at 6 hpi in G06 (log2 fold change = 2.34) and at 30 hpi in G12 (log2 fold change = 2.19) in response to Ac_Hokkaido01. Other genes reported to be involved in plant defense responses among the 125 uniquely up-regulated genes in genotype G06 are listed in Table 7.

**Table 7:** List of selected up-regulated genes involved in the defense responses to *Aphanomyces cochlioides* in the sugar beet partially resistant genotype G06

Transcript ID	Log_2_ fold change	Time point	Isolate	Name	Description
EL10Ac4g07957	6.79	30 hpi	Ac_01.7.6	Protein NDR1	Required for resistance conferred by multiple R genes
EL10Ac4g09732	2.23	30 hpi	Ac_01.7.6	Peroxidase 47	Response to stresses such as wounding, pathogen attack and oxidative stress
EL10Ac7g18203	5.70	30 hpi	Ac_01.7.6	Probable disease resistance protein At1g15890	CC-NBS-LRR protein, defense response to other organisms
EL10Ac5g10693	2.01	30 hpi	Ac_Hokkaido01	Probable LRR receptor-like serine/threonine-protein kinase At4g26540	Kinase activity
EL10Ac6g13326	3.75	30 hpi	Ac_Hokkaido01	Disease resistance protein RPM1	Triggers Hypersensitive Response (HR)
EL10Ac3g06517	1.41	6 hpi	Ac_01.7.6	Receptor-like cytosolic serine/threonine-protein kinase RBK2	Kinase activity

Some of the up-regulated genes involved in plant defense among the 475 genes that were exclusively expressed in genotype G12 are shown in Table 8.

**Table 8:** List of selected up-regulated genes as candidate defense genes in response to *Aphanomyces cochlioides* in the sugar beet partially resistant genotype G12

Transcript ID	Log_2_ fold change		Time point	Isolate	Name	Description
EL10Ac4g08863 EL10Ac2g02793	4.40 1.68	6 hpi 30 hpi		Ac_01.7.6 Ac_Hokkaido01	Putative disease resistance RPP13-like protein 1	Involved in plant defense
EL10Ac2g02511 EL10Ac5g11093	2.13 2.51 2.50 2.58	6 hpi 6 hpi 30 hpi 30 hpi		Ac_Arhill2012 Ac_Hokkaido01 Ac_01.7.6 Ac_Arhill2012	Disease resistance protein RGA2	Involved in plant defense
EL10Ac2g04100 EL10Ac2g04094	2.03 7.79	30 hpi 30 hpi		Ac_Arhill2012 Ac_Arhill2012	Putative disease resistance protein RGA3	Triggers a defense system that restricts the pathogen growth
EL10Ac2g04098	2.37	30 hpi		Ac_Arhill2012	Putative disease resistance protein At3g14460	Involved in plant defense
EL10Ac2g03197 EL10Ac8g18462	1.07	30 hpi		Ac_Hokkaido01 Ac_01.7.6 Ac_Arhill2012 Ac_Hokkaido01	MLP-like protein 43 MLP-like protein 43	Involved in defense response Involved in defense response
EL10Ac2g04536	1.55	30 hpi		Ac_Hokkaido01	Protein NtpR	Pathogenesis-related protein
EL10Ac4g07858	3.67	30 hpi		Ac_Hokkaido01	Defensin-like protein AX1	Antibiotic, antimicrobial, fungicide activity
EL10Ac6g15268	1.06	30 hpi		Ac_Hokkaido01	Protein MKS1	Regulator of plant defense response

## Discussion

In this study we hypothesized that different sets of genes are expressed in response to *A. cochlioides* invasion in susceptible and partially resistant sugar beet genotypes. Thus, we investigated changes in the transcript profiles in sugar beet breeding lines known to display different phenotypes after infection caused with the pathogen. Our aim was to identify defense-related genes that enable the plant to cope with pathogen attack during the initial stages of the infection. The major source of variation in our dataset was host genotype, with samples clustering by genotype and not by treatment. Interestingly, a recent transcriptomics investigation of the resistance responses of pea to infection by *Aphanomyces euteiches* [29] found a similar variation related to genotype, indicating that the gene expression governing resistance mechanisms against pathogen invasion strongly depends on the specific genotype.

### General differential expressed genes correlated to stress responses in sugar beet

In order to understand the generic responses induced by *A. cochlioides* infection, the function of the up-regulated genes shared between the four sugar beet breeding lines analyzed in this study was investigated. Overall, all genotypes shared some differential expressed genes, corresponding to stress-related genes. These included chalcone synthases (CHS), auxin-binding proteins, glutathione S-transferases (GSTs) and germin-like proteins (GLP).

Chalcone synthase (CHS) is a key enzyme of the flavonoid/isoflavonoid biosynthesis pathway and is known to be commonly expressed under several type of stresses including bacterial and fungal infections [30]. The expression of this gene results in the production of flavonoids and isoflavonoids with antimicrobial activity, known as phytoalexins. The accumulation of these antimicrobic metabolites in response to pathogen attacks is well established and has been described in several plant species, including sugar beet [30, 31]. KEGG analysis showed that genes involved in secondary metabolite pathways, and in particular in flavonoid and phenylpropanoid biosynthesis, underwent significant changes in both partially resistant and susceptible lines, indicating that they play a central role during the host-pathogen interaction.

The phytohormone auxin plays a central role not only in plant growth and development but also in plant immune signaling [32]. In *A. thaliana*, the auxin response pathway has been reported to be involved in resistance against oomycetes [33]. Microarray analysis of the model plant *Medicago truncatula*, performed to study the transcriptomic responses of pea (*Pisum sativum* L.) to the oomycetes *Phytophthora pisi* and *A. euteiches*, demonstrated that CHS and auxin pathways are specifically induced in response to *A. euteiches* infection [34]. The up-regulation of an auxin-responsive gene was also identified in a pea susceptible genotype in response to *A. euteiches* infection [29]. The large number of up-regulated genes corresponding to chalcone synthases and auxin-binding proteins in all sugar beet lines tested here might indicate that these pathways are also induced in sugar beet in response to the related pathogen *A. cochlioides*.

Plant glutathione S-transferases (GSTs) are ubiquitous and multifunctional enzymes whose expression is also induced under several form of abiotic and biotic stresses, including bacterial, fungal and viral infections [35]. They display a detoxification activity of toxic compounds by conjugating with glutathione, an oxidative stress attenuation and they participate in hormone transport [35]. In our study, the GO terms “detoxification” and “response to toxic substance” were enriched at certain levels in all samples at 6 hpi and 30 hpi and in response to different *A. cochlioides* isolates. The role of GSTs during infection by necrotrophic pathogens has been investigated in other pathosystems. For example, a proteomic study revealed an accumulation of catalase 3 and multiple GSTs in *A. thaliana*, following infection with the necrotrophic fungus *Botrytis cinerea*, demonstrating the importance of an antioxidant system in defense against the fungus [36]. Catalase 1 and GSTs are known to protect host cells against reactive oxygen species. The breakdown of hydrogen peroxide (H_2_O_2_) by conversion into molecular oxygen and water is mediated by catalase 1 [37]. Therefore, the detoxification activity that undergoes changes during infection with *A. cochlioides* could be a consequence of the accumulation of H_2_O_2_ formed during plant-pathogen interactions.

Germin-like proteins (GLPs) are ubiquitous glycoproteins reported in both higher and lower plants, characterized by several biochemical properties such as oxalate oxidase (OxO) and superoxide dismutase (SOD) activities [38]. They are known to be involved in plant defense to biotic and abiotic stresses and several studies have demonstrated their role in plant basal resistance to fungal pathogens [39]. GLPs also belong to the pathogenesis-related protein family and are part of plant basal resistance [40]. Many of them are localized to the plant cell wall and play a role in cell wall reinforcement by participating in the cross-linking of cell wall components [41]. The germin-like protein *BvGLP-1* gene from sugar beet, which exhibits sequence similarity to other plant GLPs, is highly up-regulated in resistant plants during nematode infection. The over-expression of *BvGLP-1* in *A. thaliana* constitutively activated the expression of a subset of plant defense-related proteins, inducing resistance against the pathogens *Verticillium longisporum* and *R. solani*, by elevating the levels of H_2_O_2_ [42]. A novel GLP isolated from cotton (*Gossypium hirsutum* L.), *GhABP19*, was demonstrated to be involved in the defense against the fungal pathogens *V. dahliae* and *Fusarium oxysporum* [43].

The increased expression of these genes in both partially resistant and susceptible sugar beet lines in our study, indicates that they play an important role in constitutive defense responses to stress conditions and invading pathogens. However, the inhibition of the pathogen growth depends on the ability of the plant to recognize and identify the invader and its secreted molecules. Therefore, the pathogen could be able to overcome this first barrier of defense and to suppress downstream host defenses in the susceptible hosts.

### Enriched GO biological processes and pathways in partially resistant and susceptible sugar beet genotypes in response to *A. cochlioides*

Changes in genes associated with photosynthesis (specifically the light reaction), following the infection were the most overrepresented in the two susceptible sugar beet lines, especially at 30 hpi. It is known that photosynthesis is not only affected by changes in the environmental conditions and abiotic stresses but also by pathogen invasion [44]. However, the mechanisms that mediate these changes after pathogen attacks remain unclear. A possible reason could be an increased demand for assimilates from the pathogen but changes in photosynthesis and other assimilatory metabolisms are also proposed to be a plant strategy to invest energy in coping with the pathogen invasion [45]. Alterations in photosynthesis, but in the dark reaction, were observed also in the partially resistant genotype G06 at 6 hpi, suggesting that this genotype might promptly re-direct energy to defense responses. KEGG analysis showed that several DEGs in the susceptible lines were part of photosynthetic pathways (i.e., carbon fixation, photosynthesis). In addition to changes in photosynthetic activities, which appeared to be predominant in genotype G01, other responses were triggered in genotype G17, which include genes with GO terms related to interspecies interactions and responses to fungus/another organism. Genes involved in defense responses were also up-regulated in G17 at 30 hpi, indicating that host defense responses are elicited in this genotype at the initial phase of the infection and are probably overcome at later stages by the pathogen.

The overall responses to *A. cochlioides* infection between the two partially resistant genotypes G06 and G12 appeared to be diverse. The most enriched GO terms in genotype G06 were associated to ROS metabolic process, in particular H_2_O_2_ and to detoxification and response to toxic substances. H_2_O_2_ is known to play multiple roles in plant responses to pathogens. It directly limits the viability and the spread of the pathogen, it induces the production of phytoalexins, it is involved in the localized plant cell death during the hypersensitive response, it acts as signal to the systemic acquired resistance and lastly it induces the expression of defense genes [46]. Changes in genes associated with the metabolism of H_2_O_2_ suggest that it might have a central role in regulating defense responses to *A. cochlioides*, while the enrichment of GO terms associated to detoxification could be interpreted as a consequence of the high accumulation of H_2_O_2_ which, at high levels, is toxic and can cause damage to cell structures [47]. Similar responses were observed in the susceptible genotype G17 at the earliest time point. These results might indicate that similar mechanisms are activated in response to the pathogen invasion in both the partially resistant and the susceptible genotypes, but the interaction with the pathogen subsequently leads to different reactions in the two hosts. In the other partially resistant genotype G12, the most interesting GO biological process was cell wall organization. The cell wall acts as physical barrier to prevent pathogen ingress into the cell and, in response to an attack, plants can deposit polymers to strengthen the cell wall [48]. Changes in genes associated to H_2_O_2_ metabolism were also observed in genotype G12 at the earliest time point (6 hpi), confirming that during the infection process changes in the metabolism of this ROS are induced. H_2_O_2_ is also required for peroxidase-dependent lignification and for protein cross-linking in the cell wall, making the cell more resistant to cell wall-degrading enzymes [48]. Therefore, cell wall reorganization could be a defense mechanism triggered in genotype G12 to attempt to physically prevent penetration by the pathogen. In addition, the GO biological processes “response to chitin”, “response to other organism”, “interspecies interaction” and “defense response” were enriched in genotype G12 at 30 hpi, indicating the induction of specific responses following the detection of the pathogen.

### Responses in specific genotype-isolate-time point interactions

The observed similarities and differences in GO biological processes across different genotypes in response to different *A. cochlioides* isolates highlight complex interactions between plant genotypes and pathogen isolates. Different genotypes exhibit unique sets of gene expression changes in various biological processes, likely shaped by both genetic variation and pathogen-specific factors. This suggests that each genotype has a specialized defense approach, which may provide them with adaptive advantages depending on the type of pathogen or the environmental stress they encounter. Despite these differences, certain processes were shared across genotypes. Interestingly, when looking at specific genotype-isolate interactions, most of the common biological processes were shared between susceptible genotypes with partially resistant genotypes (i.e., G01 with G06 and G17 with G12), while common responses among the two susceptible and the two partially resistant genotypes were less frequent. For instance, pathogen-specific responses were shared between G12 and G17. Ac_01.7.6 triggered H_2_O_2_-related processes in both G12 and G17, and Ac_Hokkaido01 caused responses related to toxin catabolism and detoxification in G12 and G17, indicating that these isolates may induce the activation of H_2_O_2_-related defense mechanisms and detoxification pathways in these genotypes. Similarly, phenylpropanoid, secondary metabolites and lignin biosynthetic processes were over-represented in both G01 and G06 in response to Ac_Arhill2012. Phenylpropanoids serve as precursors to antimicrobial compounds, including flavonoids, stilbenes, and tannins. Moreover, phenylpropanoids play a role in signaling pathways that activate broader immune responses [49]. Secondary metabolites produced through phenylpropanoid pathways, including phytoalexins and terpenoids, have direct antimicrobial properties, making them critical for pathogen defense [50]. This pathway is also involved in the synthesis of lignin which strengthens the cell wall, creating a physical barrier that impedes pathogen invasion [51]. The over-representation of these pathways in G01 and G06 in response to Ac_Arhill2012 and at an early time point (6 hpi) suggests that these genotypes quickly activate both structural defenses and the production of biochemical compounds to inhibit pathogen growth and signal further immune responses. Moreover, common defense mechanisms like ROS regulation and photosynthesis modulation were seen across multiple genotypes, despite the fact that they were triggered by different isolates or at different time points. This can be attributed to conserved plant defense mechanisms that are commonly triggered during pathogen attack.

### Differentially expressed genes involved in host plant resistance towards *A. cochlioides*

Resistance (R) proteins act as primary receptors of pathogen effectors or have an indirect effect in the recognition process [52] and initiate signal transduction pathways that result in the expression of disease resistance through activation of the hypersensitive response (HR) and other responses [53]. Among the up-regulated genes observed in the two partially resistant genotypes but not expressed in the susceptible lines, 18 genes were in common between G06 and G12. One of these genes corresponds to a gene annotated as “Probable disease resistance protein At1g15890”, that belongs to the CC-NBS_LRR class of disease resistance proteins. Another of these genes is annotated as mitogen-activated protein kinase 4 (MKK4), which is involved in the second phase of H_2_O_2_ generation during HR. However, most of the up-regulated genes uniquely expressed in the partially resistant lines were genotype-specific. In addition, each genotype shared only a small proportion of DEGs in response to the three different isolates and showed unique up-regulated genes to specific isolates, suggesting that these two genotypes have distinct defense mechanisms to combat pathogen invasion and that the responses vary depending on the pathogen isolate they encounter. In genotype G06 we observed the up-regulated expression at 30 hpi of genes annotated as homologues of the *A. thaliana* genes NDR1, which is linked to many R genes and is required for the establishment of both HR and systemic acquired resistance (SAR) [54], and the NBS-LRR resistance gene RPM1, that, for example, leads to the restriction of *Pseudomonas syringae* growth through HR [55]. A homologous gene of NDR1 was also up-regulated in a susceptible pea genotype in response to *A. euteiches* infection at 48 hpi [29] Three putative disease resistance proteins (RPP13-like protein 1, RGA3 and At3g14460) and the disease resistance protein RGA2 were up-regulated in genotype G12. The RPP13 resistance gene family was first discovered in *A. thaliana* and confers resistance to downy mildew caused by the oomycete *Hyaloperonospora parasitica* [56]. It functions independently of NDR1 and EDS1 and does not require the accumulation of salicylic acid for activation [57]. AtLRRAC1 (At3g14460) is a LRR class R gene with adenylyl cyclase activity which is cAMP dependent and promotes defenses against biotrophic and hemibiotrophic pathogens in *A. thaliana* but has not been shown to be active against necrotrophic pathogens. It is therefore hypothesized to be involved in early PAMP Triggered Immunity (PTI) signaling rather than acting as a classical R-gene that recognizes specific effectors [58]. Our results therefore suggest that the two genotypes might initiate an immune response via so-called effector-triggered immunity (ETI), upon R genes recognition of *A. cochlioides* effector(s). However, more generalized, earlier acting PTI responses may also be important for resistance to *A. cochlioides.* Since it is now well known that these pathways are convergent and potentiate one another [59], further research is required to untangle the precise timing and activation of these responses in sugar beet. Furthermore, multiple different pathways representing both NDR1 dependent and independent mechanisms may be activated within the defense responses of the different genotypes. It is possible that genotype G06 is dependent on NDR1 pathways, whilst G12 may use NDR1-independent pathways, although further research is required to test this hypothesis. In addition, an up-regulated gene annotated as MLP-like protein 43 was identified in genotype G12 at 30 hpi. Besides their role in drought and salt tolerance, Major Latex-like Proteins (MLPs) are also known to induce resistance against pathogens [60]. Three MLPs homologous genes have been reported to be highly expressed in sugar beet roots of partially resistant genotypes, 5 days after inoculation with the soil-borne basidiomycete *R. solani* [15]. Their role in plant defense has also been observed in response to other fungal pathogens, namely *V. dahlia* [61] and *Alternaria brassicicola* [62] and to the soil-borne plasmodiophorid *Plasmodiophora brassicae* [63]. Although their function remains to be investigated, the expression of these genes during the infection process indicates that they play an important role in regulating plant resistance mechanisms and, therefore, that the MLP-like protein 43 could contribute to defense responses to *A. cochlioides* in the sugar beet partially resistant genotype. Another gene annotated as pathogenesis-related protein (PR), NtpR, was expressed in genotype G12 at 30 hpi. A previous study where the *NtPR-Q* gene has been over-expressed in tobacco (*Nicotiana tabacum* L.) has shown the role of this gene in inducing expression of defense-related genes and in defending tobacco against the pathogen *Ralstonia solanacearum*.

In addition, several TFs were identified among the DEGs of the different genotypes. Some of them are known to be associated with plant defense responses. Among the up-regulated genes, we detected several ethylene-responsive TFs (ERF8, ERF095, ERF109 and RAP2-3) which bind the GCC-box promoter element of pathogenesis-related (PR) genes, whose expression is up-regulated following pathogen attack and WRKY40 and WRKY70, belonging to the WRKY family, which regulate the defense responses to bacteria, fungi and oomycetes. A TF belonging to the HB family, OCP3, was also detected in the partially resistant genotype G12. OCP3 has been reported to modulate NPR1-mediated jasmonic acid-induced defenses in *A. thaliana* [64].

## Conclusions

Our data provides insights into the responses of sugar beet to infection by *A. cochlioides.* Global reactions to *A. cochlioides* infection consist of induction of secondary metabolites and flavonoid biosynthesis and an up-regulation of chalcone synthases (CHS), auxin-binding proteins, glutathione S-transferases (GSTs) and germin-like proteins (GLP). Changes in photosynthetic activity are triggered in response to the pathogen attack in the susceptible lines, while changes in H_2_O_2_ metabolism, detoxification processes and cell wall reorganization were the most represented in the two partially resistant lines. Specific genotype-isolate-time point interactions showed distinct and shared GO categories, suggesting that different genotypes employ both universal defense mechanisms and specialized responses tailored to specific isolates at different times. Furthermore, candidate genes involved in plant defense responses and immunity were identified in the set of up-regulated genes uniquely expressed in the partially resistant genotypes. Our data suggest that both PTI and ETI responses may be important components of immune responses to *A. cochlioides* and that these responses vary by genotype. Future studies on these genes will elucidate their role in the defense mechanisms in response to *A. cochlioides* infection.

## Electronic supplementary material

Below is the link to the electronic supplementary material.

Supplementary Material 1

## Data Availability

The datasets generated and analyzed during the current study are available in the NCBI repository (https://www.ncbi.nlm.nih.gov/nuccore/), under Bioproject “PRJNA1072272”. These data are also available at the following link: https://www.ncbi.nlm.nih.gov/sra/PRJNA1072272 with project metadata available here: https://www.ncbi.nlm.nih.gov/bioproject/PRJNA1072272.

## Abbreviations

CC-NBS: Coiled-Coil domain- Nucleotide-Binding Site domain
LRR: Leucine-Rich Repeat
CHS: Chalcone Synthases
DE: Differential Expression
DEGs: Differentially Expressed Genes
ERF: Ethylene Response Factor
GLPs: Germin-Like Proteins
GO: Gene Ontology
GSTs: Glutathione S-Transferases
HB: Homeobox
HR: Hypersensitive Response
KEGG: Kyoto Encyclopedia of Genes and Genomes
MAPK: Mitogen-Activated Protein Kinases
MLP: Major Latex Protein
OxO: Oxalate Oxidase
PCA: Principal Component Analysis
QTLs: Quantitative Trait Loci
R: Resistant
ROS: Reactive Oxygen Species
SAR: Systemic Acquired Resistance
SOD: Superoxide Dismutase
TFs: Transcription Factors
