# Supplementary material for: Transcriptome analysis of sugar beet in response to the pathogenic oomycete Aphanomyces cochlioides
Source: BMC Plant Biol. 2024 Dec 18;24:1177. doi: 10.1186/s12870-024-05910-y (PMC11653986; doi:10.1186/s12870-024-05910-y)
Supplement: Supplementary file 1 — Supplementary Material 1 [file 12870_2024_5910_MOESM1_ESM.docx]

**Supplementary Information Rossi et al BMC Plant Biology**


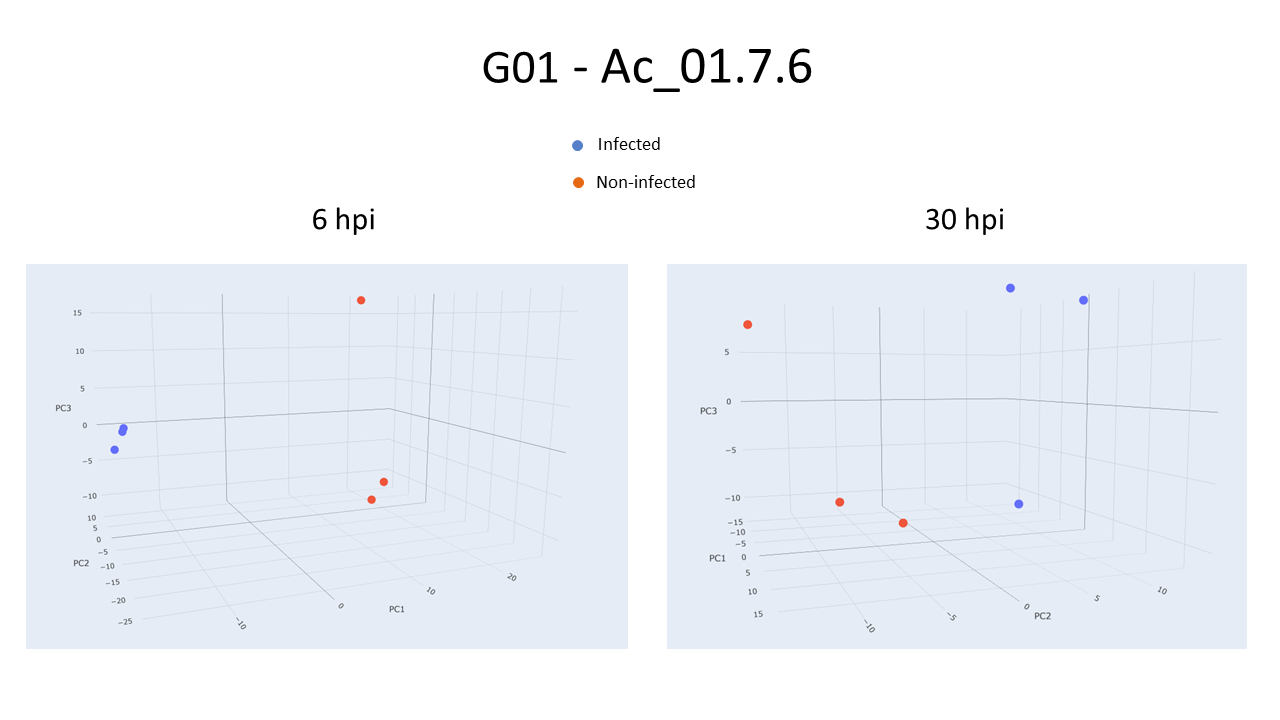
**Additional file 1** 3D PCA plot of samples belonging to genotype G01 inoculated with the *A. cochlioides* isolate Ac_01.7.6 and their respective non-inoculated samples at 6 hpi (left) and 30 hpi (right)


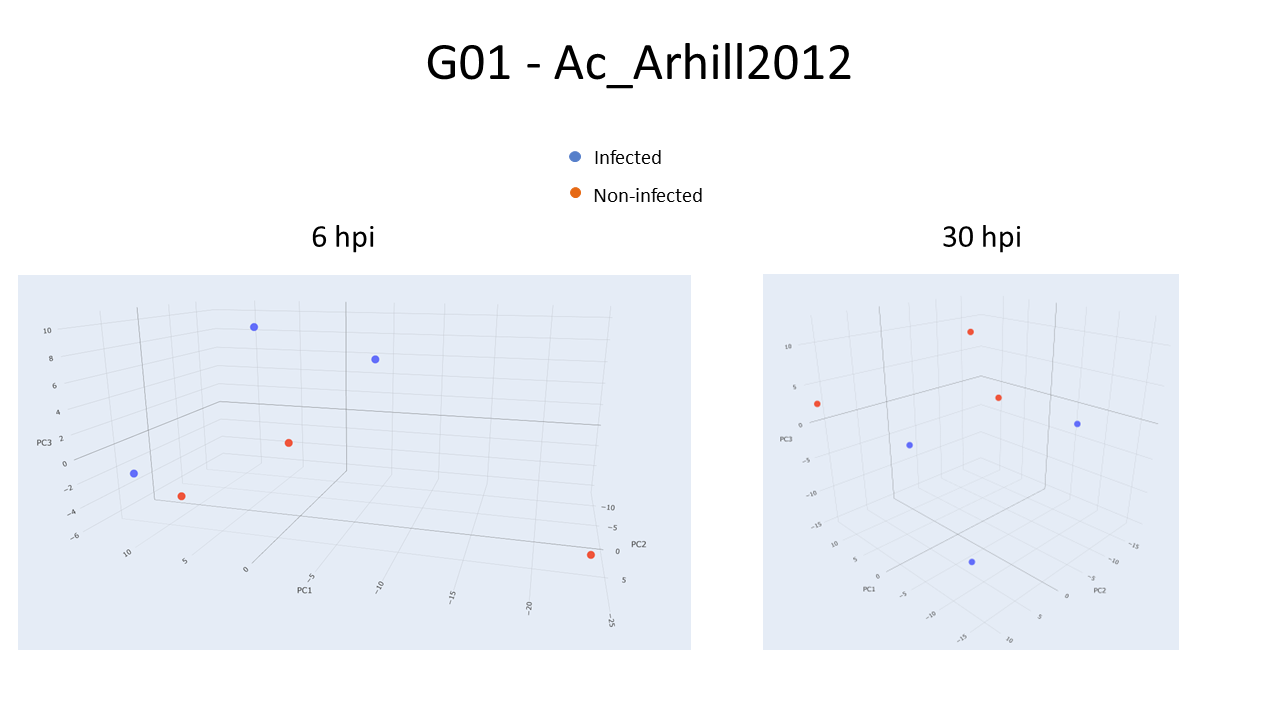
**Additional file 2** 3D PCA plot of samples belonging to genotype G01 inoculated with the *A. cochlioides* isolate Ac_Arhill2012 and their respective non-inoculated samples at 6 hpi (left) and 30 hpi (right)


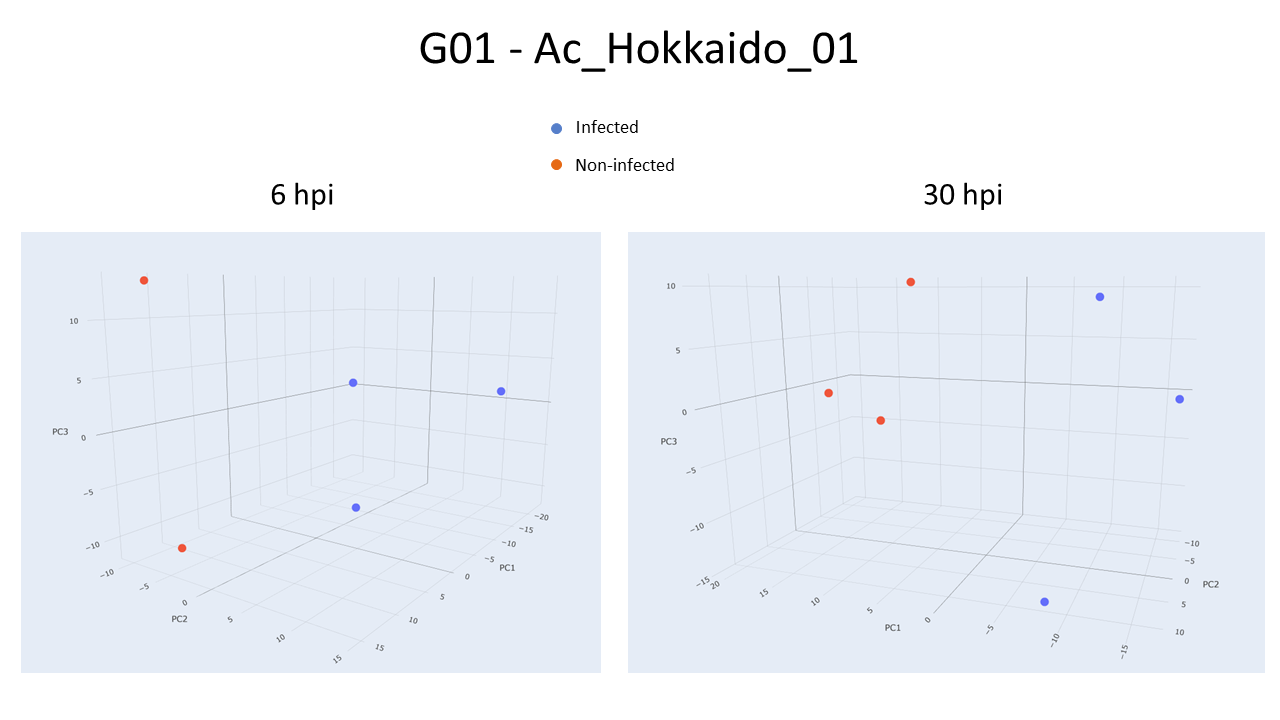


**Additional file 3** 3D PCA plot of samples belonging to genotype G01 inoculated with the *A. cochlioides* isolate Ac_Hokkaido_01 and their respective non-inoculated samples at 6 hpi (left) and 30 hpi (right)


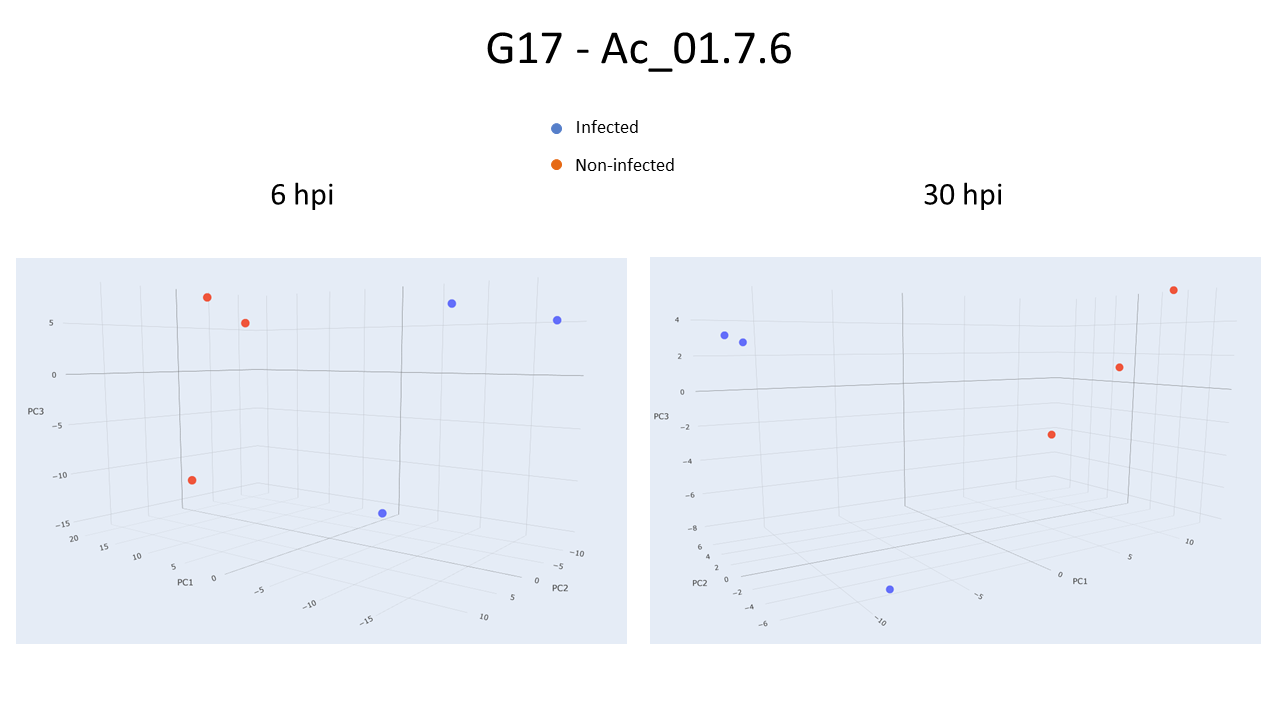
 **Additional file 4** 3D PCA plot of samples belonging to genotype G17 inoculated with the *A. cochlioides* isolate Ac_01.7.6 and their respective non-inoculated samples at 6 hpi (left) and 30 hpi (right)


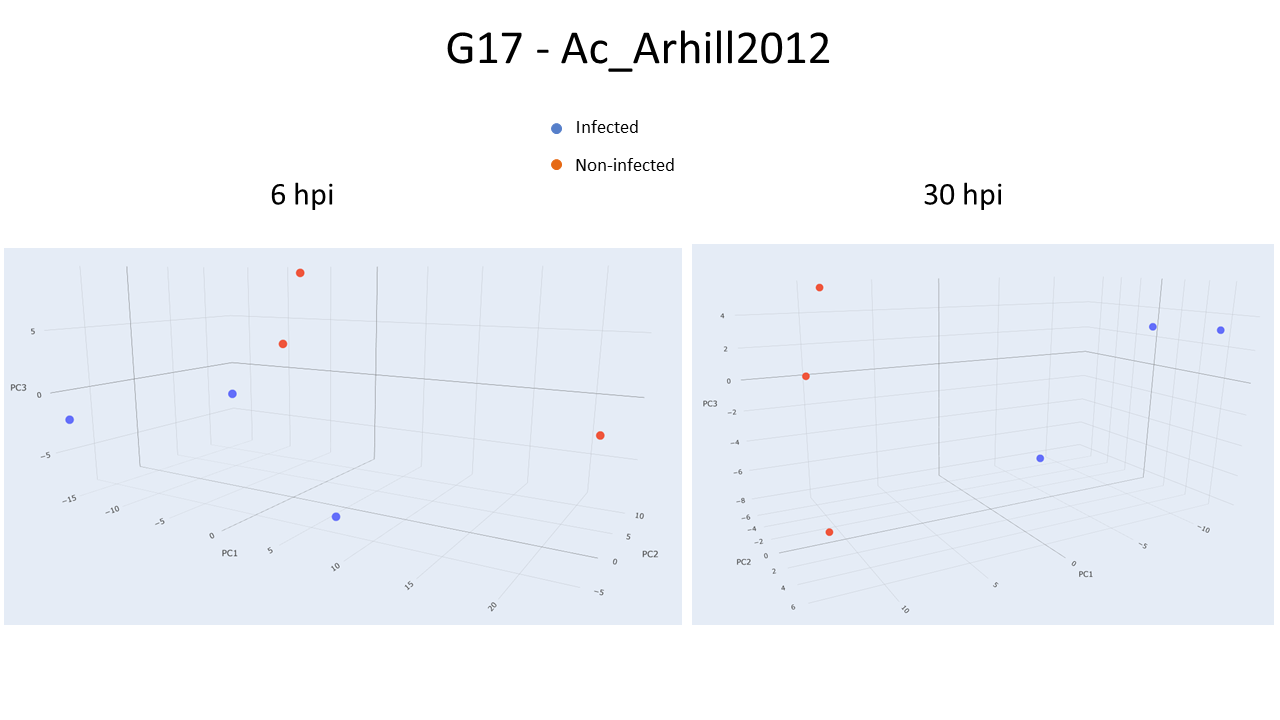
 **Additional file 5** 3D PCA plot of samples belonging to genotype G17 inoculated with the *A. cochlioides* isolate Ac_Arhill2012 and their respective non-inoculated samples at 6 hpi (left) and 30 hpi (right)


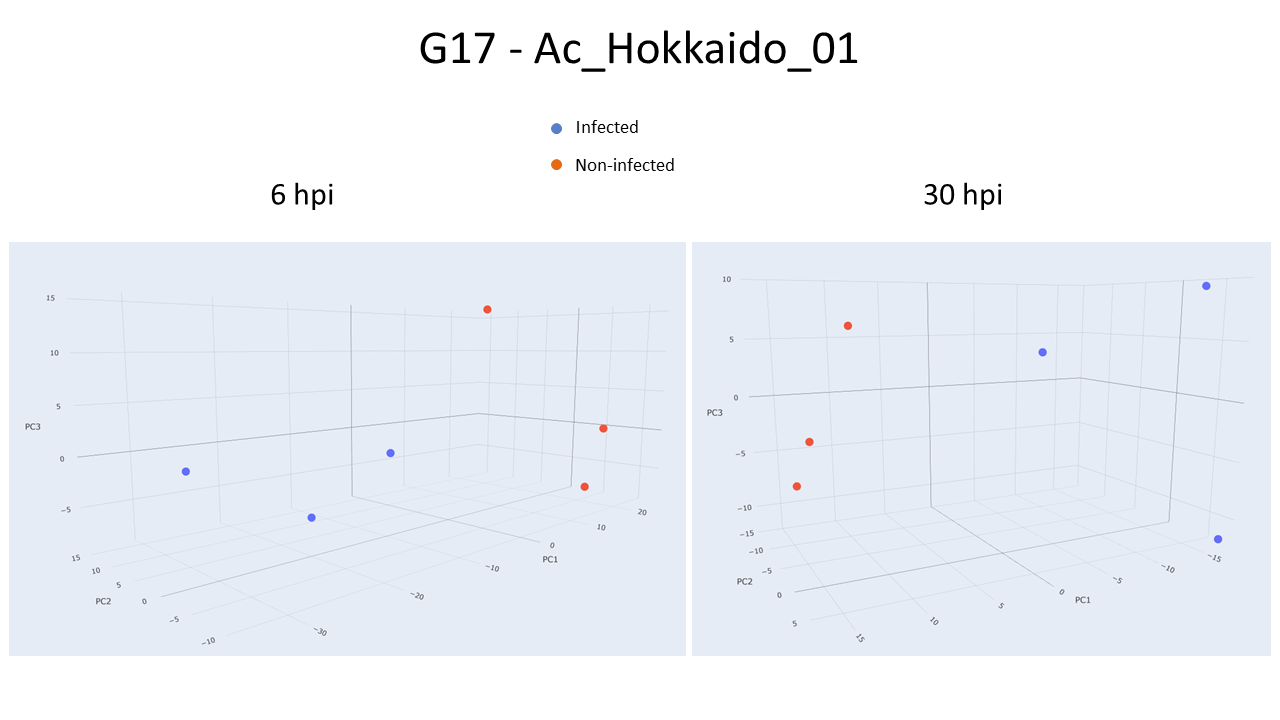


**Additional file 6** 3D PCA plot of samples belonging to genotype G17 inoculated with the *A. cochlioides* isolate Ac_Hokkaido_01 and their respective non-inoculated samples at 6 hpi (left) and 30 hpi (right)


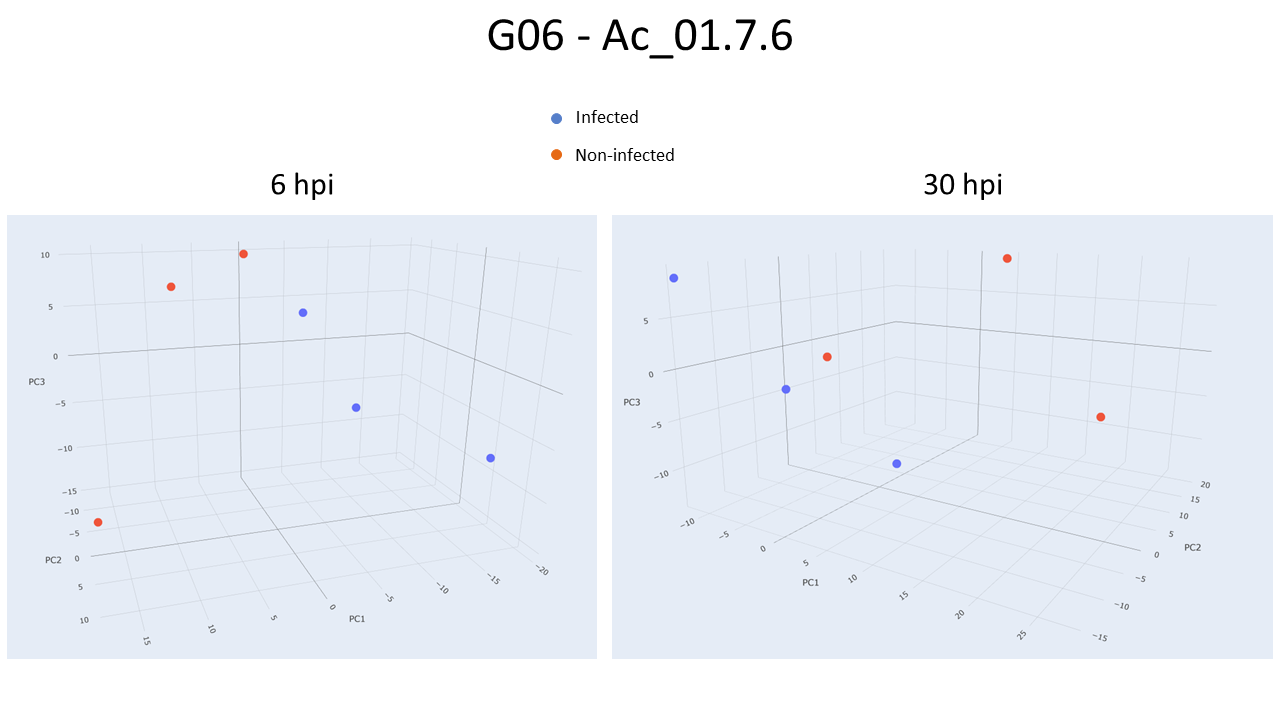
 **Additional file 7** 3D PCA plot of samples belonging to genotype G06 inoculated with the *A. cochlioides* isolate Ac_01.7.6 and their respective non-inoculated samples at 6 hpi (left) and 30 hpi (right)


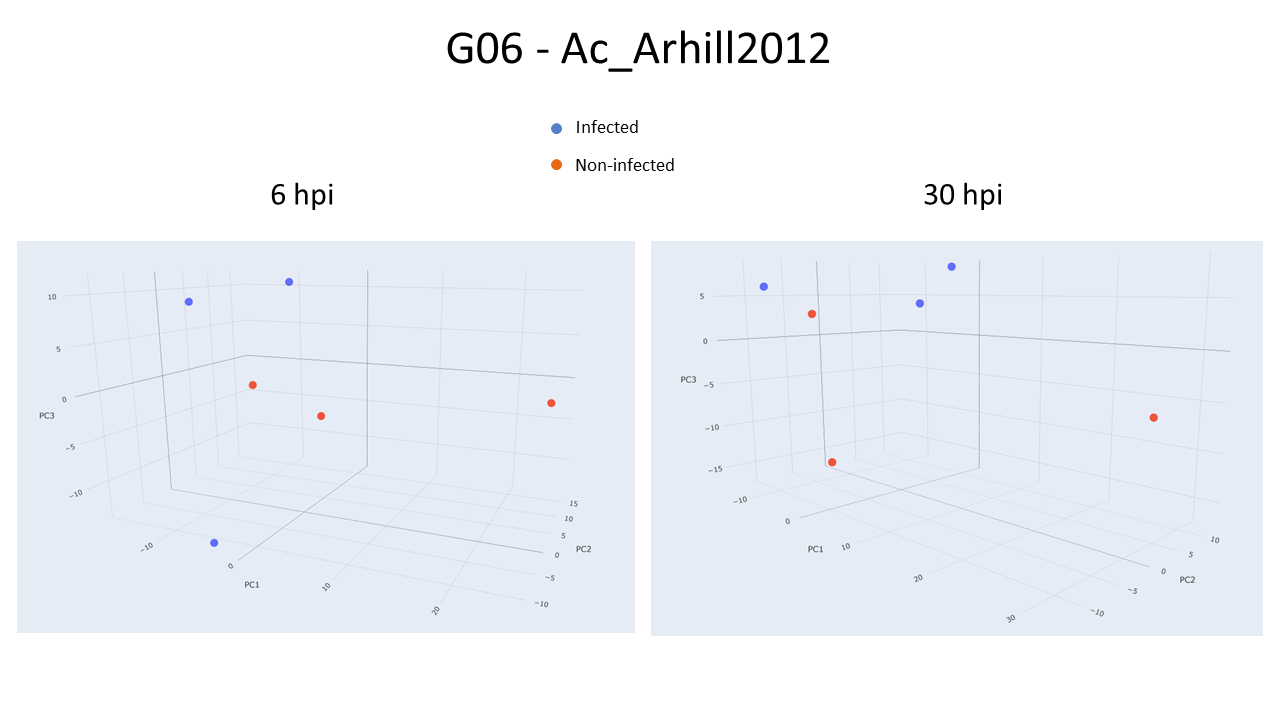
**Additional file 8** 3D PCA plot of samples belonging to genotype G06 inoculated with the *A. cochlioides* isolate Ac_Arhill2012 and their respective non-inoculated samples at 6 hpi (left) and 30 hpi (right)


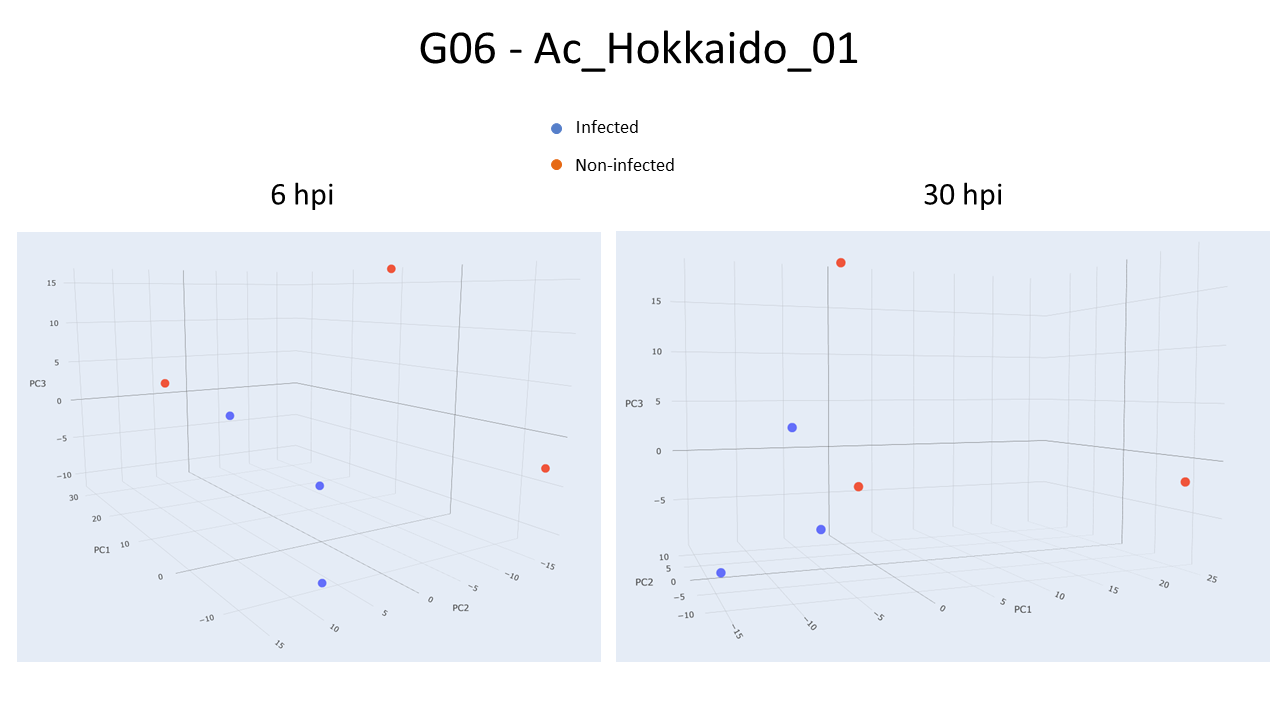
 **Additional file 9** 3D PCA plot of samples belonging to genotype G06 inoculated with the *A. cochlioides* isolate Ac_Hokkaido_01 and their respective non-inoculated samples at 6 hpi (left) and 30 hpi (right)


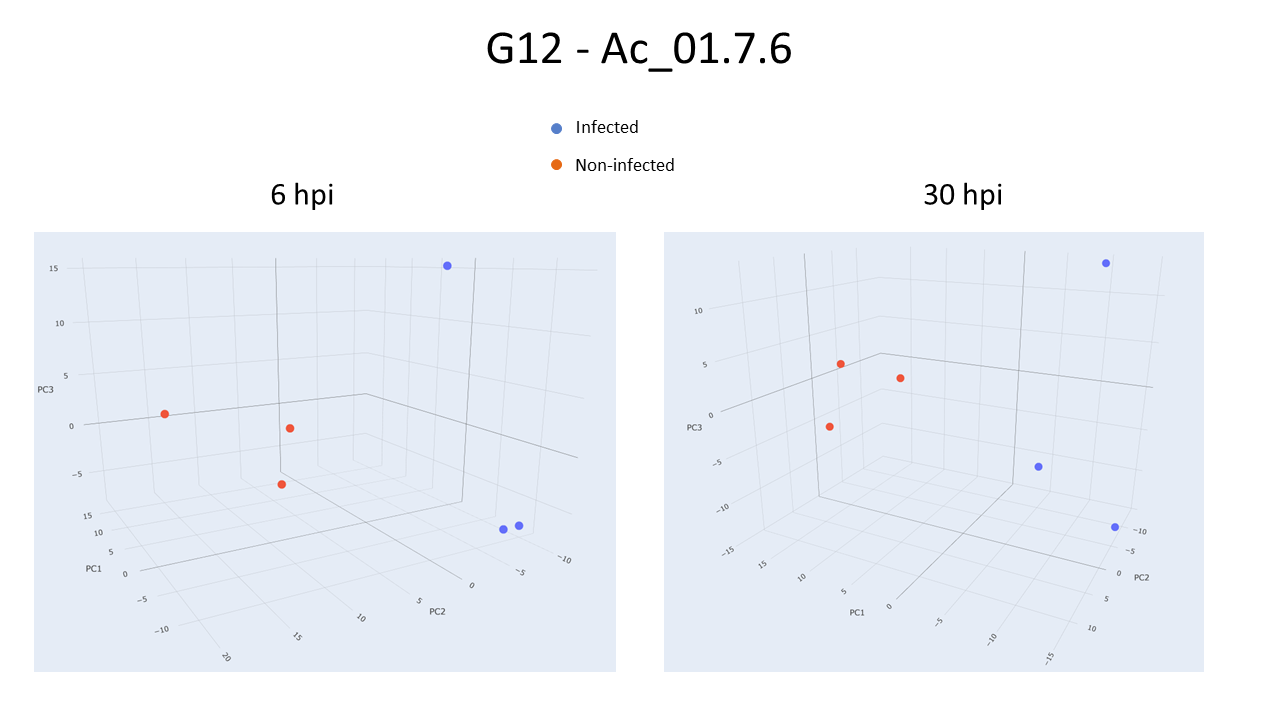
 **Additional file 10** 3D PCA plot of samples belonging to genotype G12 inoculated with the *A. cochlioides* isolate Ac_01.7.6 and their respective non-inoculated samples at 6 hpi (left) and 30 hpi (right)


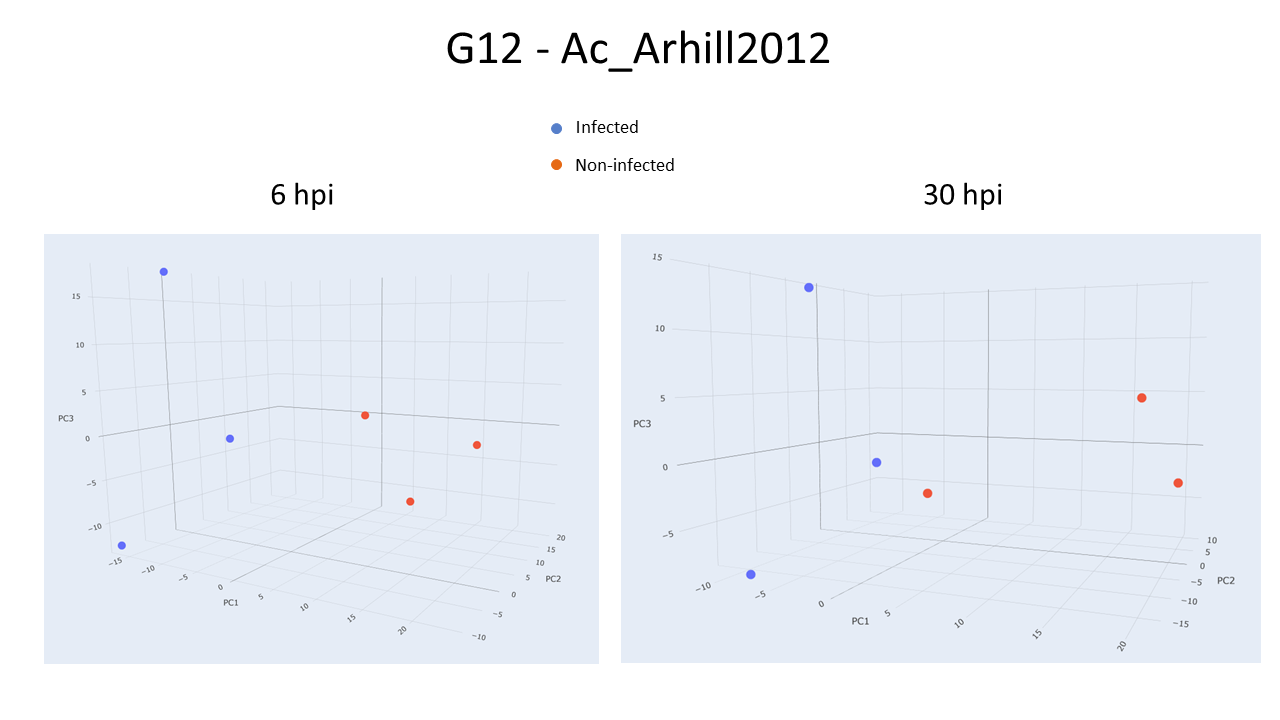
 **Additional file 11** 3D PCA plot of samples belonging to genotype G12 inoculated with the *A. cochlioides* isolate Ac_Arhill2012 and their respective non-inoculated samples at 6 hpi (left) and 30 hpi (right)


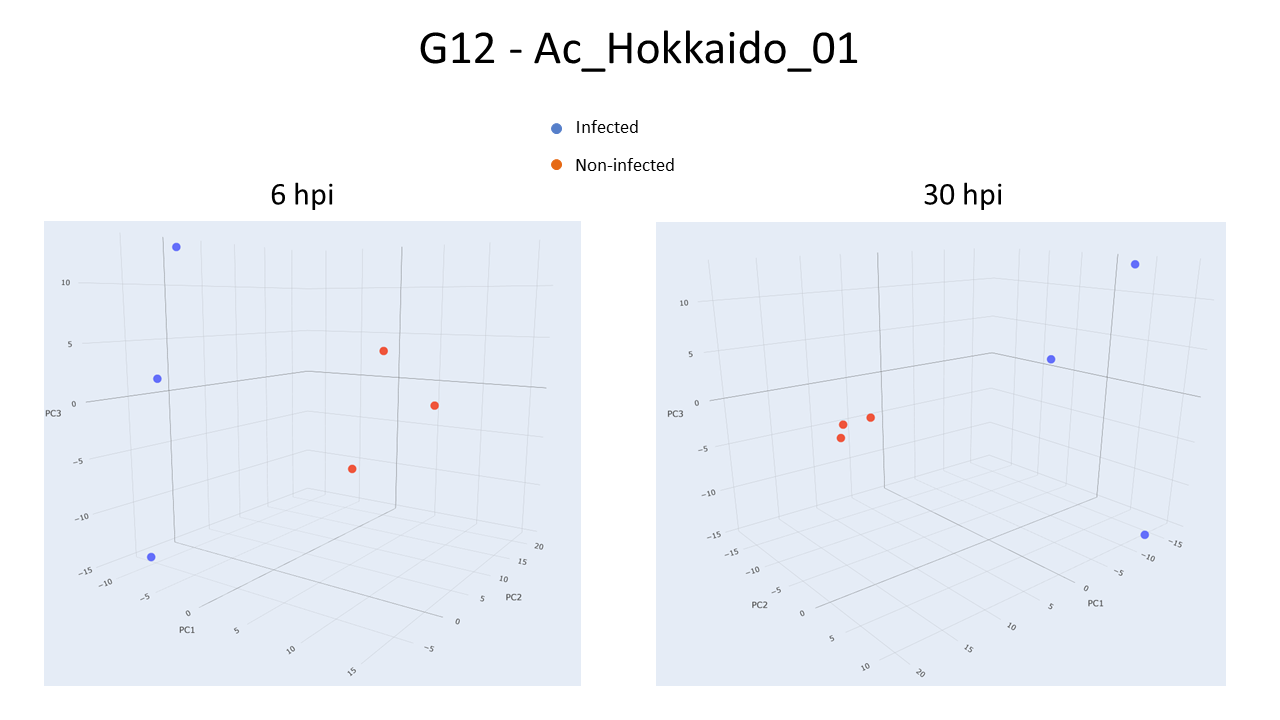
 **Additional file 12** 3D PCA plot of samples belonging to genotype G12 inoculated with the *A. cochlioides* isolate Ac_Hokkaido_01 and their respective non-inoculated samples at 6 hpi (left) and 30 hpi (right)


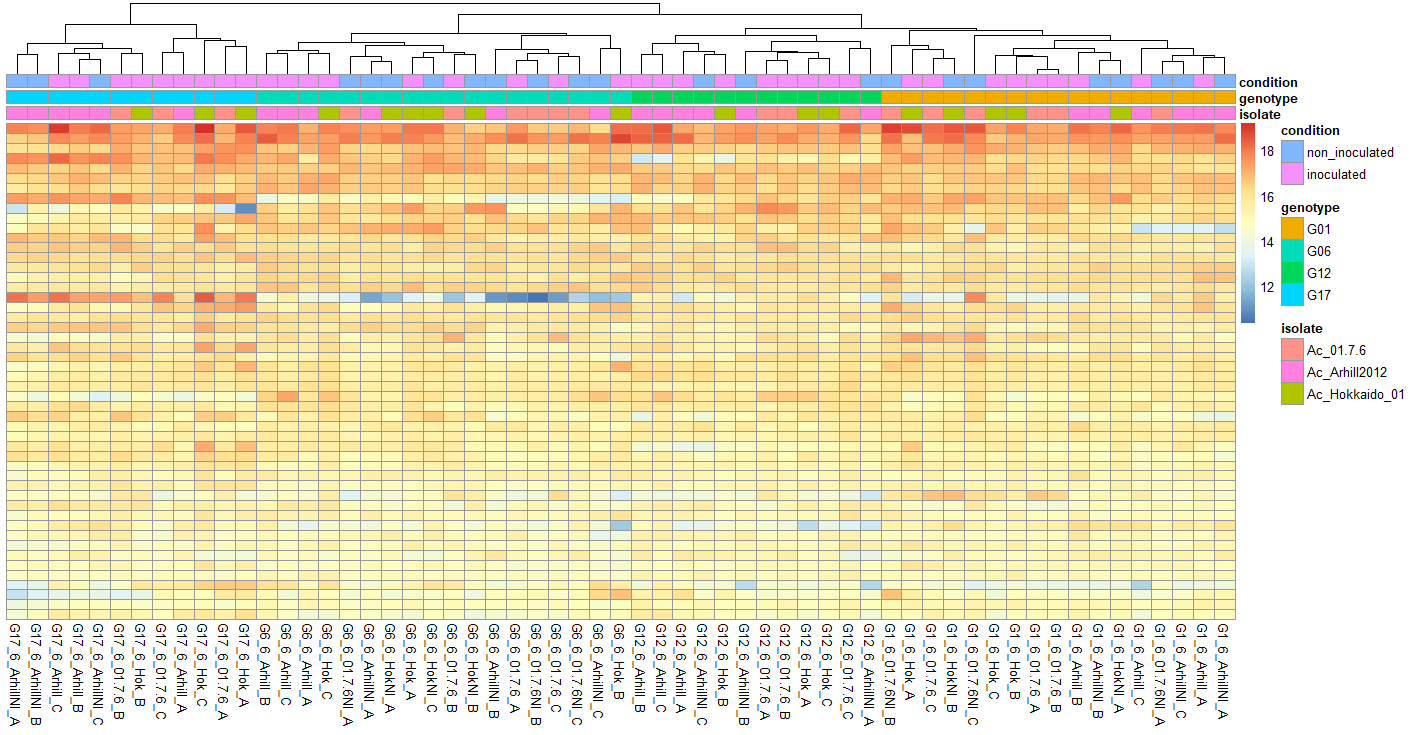


**Additional file 13** Heatmap of the of top 50 differentially expressed genes at 6 hpi in sugar beet samples inoculated with different *A. cochlioides* isolates and non-inoculated samples at the same time point. Each row represents one of the differentially expressed genes (DEGs) and each column represents a sugar beet sample from the inoculated and non-inoculated dataset

**
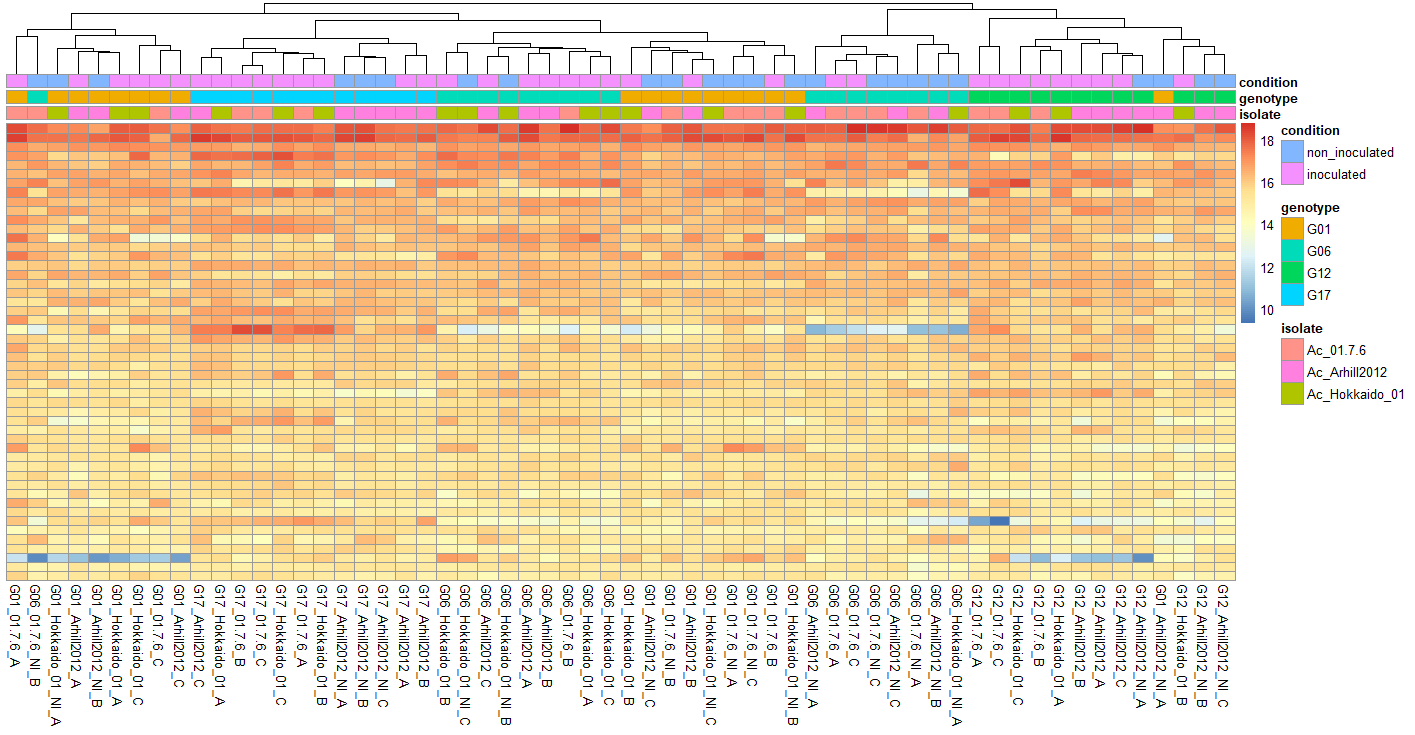
**

**Additional file 14** Heatmap of the of top 50 differentially expressed genes at 30 hpi in sugar beet samples inoculated with different *A. cochlioides* isolates and non-inoculated samples at the same time point. Each row represents one of the differentially expressed genes (DEGs) and each column represents a sugar beet sample from the inoculated and non-inoculated dataset


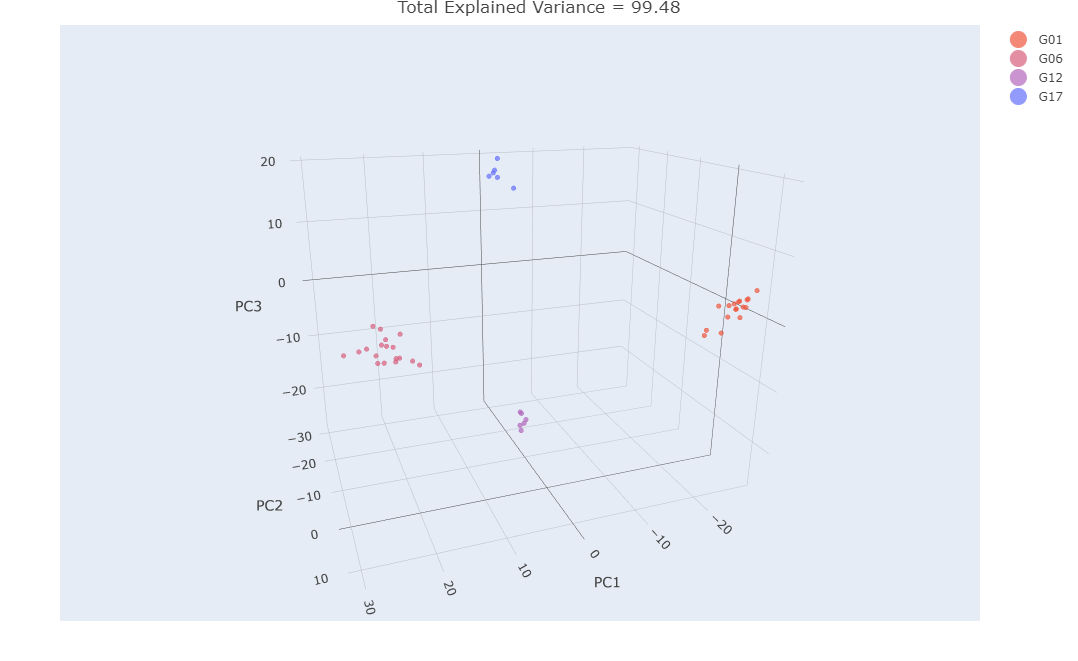
 **Additional file 15** 3D PCA plot of sugar beet non-inoculated samples belonging to different genotypes
